# Transcriptome of Small Regulatory RNAs in the Development of the Zoonotic Parasite *Trichinella spiralis*

**DOI:** 10.1371/journal.pone.0026448

**Published:** 2011-11-01

**Authors:** Xiaolei Liu, Yanxia Song, Huijun Lu, Bin Tang, Xianyu Piao, Nan Hou, Shuai Peng, Ning Jiang, Jigang Yin, Mingyuan Liu, Qijun Chen

**Affiliations:** 1 Key Laboratory of Zoonosis, Ministry of Education, Institute of Zoonosis, Jilin University, Changchun, China; 2 Laboratory of Parasitology, Institute of Pathogen Biology, Chinese Academy of Medical Sciences/Peking Union Medical College, Beijing, China; 3 Biological Therapy Center, The First Affiliated Hospital, Jilin University, Changchun, China; Oregon State University, United States of America

## Abstract

**Background:**

*Trichinella spiralis* is a parasite with unique features. It is a multicellular organism but with an intracellular parasitization and development stage. *T. spiralis* is the helminthic pathogen that causes zoonotic trichinellosis and afflicts more than 10 million people worldwide, whereas the parasite's biology, especially the developmental regulation is largely unknown. In other organisms, small non-coding RNAs, such as microRNAs (miRNA) and small interfering RNAs (siRNA) execute post-transcriptional regulation by translational repression or mRNA degradation, and a large number of miRNAs have been identified in diverse species. In *T. spiralis*, the profile of small non-coding RNAs and their function remains poorly understood.

**Methodology and Principal Findings:**

Here, the transcriptional profiles of miRNA and siRNA in three developmental stages of *T. spiralis* in the rat host were investigated, and compared by high-throughput cDNA sequencing technique (“RNA-seq”). 5,443,641 unique sequence tags were obtained. Of these, 21 represented conserved miRNAs related to 13 previously identified metazoan miRNA families and 213 were novel miRNAs so far unique to *T. spiralis*. Some of these miRNAs exhibited stage-specific expression. Expression of miRNAs was confirmed in three stages of the life cycle by qRT-PCR and northern blot analysis. In addition, endogenous siRNAs (endo-siRNAs) were found mainly derived from natural antisense transcripts (NAT) and transposable elements (TE) in the parasite.

**Conclusions and Significance:**

We provide evidence for the presence of miRNAs and endo-siRNAs in *T. spiralis*. The miRNAs accounted for the major proportion of the small regulatory RNA population of *T. spiralis*, while fewer endogenous siRNAs were found. The finding of stage-specific expression patterns of the miRNAs in different developmental stages of *T. spiralis* suggests that miRNAs may play important roles in parasite development. Our data provide a basis for further understanding of the molecular regulation and functional evolution of miRNAs in parasitic nematodes.

## Introduction

Parasites of the genus *Trichinella* are a group of pathogens with diverse biological and pathological features. Phylogenetic analysis of the mitochondrial DNA of the species identified so far indicated that there are two genetic clades that form unique monophyletic lineages [1]. One clade is represented by *T. pseudospiralis*, which does not capsulate in the muscle cells, while the other clade is represented by *T. spiralis* while does encapsulate in muscle cells, named nurse cells, with a parasite inside the cell, surrounded by a sick layer of collagen. Parasites of *Trichinella* genus are unique intracellular pathogens. Interestingly, their entire life cycle can be completed within an animal, and trichinellosis in human and other mammals was caused through the ingestion of parasite-contaminated meat. This is a typical zoonotic disease that affects more than 10 million people worldwide [2].

After being ingested with the infected muscle tissue, L1 larvae are released and activated in the small intestine, enter the epithelial layer and undergo four times of moultings before maturation into adult worms. Mating is initiated on day 2 after infection and newborn larvae are released by the females into the mucosa as early as 4 days post infection (dpi) [3]. The larvae migrate through the lymphatic and blood vessels, invade striated muscle cells and develop into the infective Ll stage over a period of 2–3 weeks to the next host which complete the life cycle [4]. Thus, unlike other nematodes, *T. spiralis* does not have an embryonic developmental stage in the egg, which differs markedly in biological and molecular characteristics from other nematodes, especially the well-characterized free-living worm *Caenorhabditis elegans*. Intriguingly, compared to other nematodes, *T. spiralis* has a much smaller genome with 64 Mb in nuclear DNA, which contains ≈15,808 genes [5]. The availability of genome sequence information has made it possible to dissect parasite biology.

Small non-coding RNAs (sncRNAs) are a large group of small endogenous RNAs that have been widely identified in animals [6], plants [7], fungi [8], [9] and some viruses [10]. They are generally 21–23 nucleotides in length, which guide various processes involving sequence-specific silencing through chromatin modification, mRNA degradation, and translational repression [11]–[13]. Based on their origins, structures, associated proteins and biological roles, sncRNAs are divided into three general categories: microRNAs (miRNAs), endogenous small interfering RNAs (endo-siRNAs), and piwi-interacting RNAs (piRNAs) [14]. MiRNA and endo-siRNAs have been discovered in diverse animals and plants and fungi [8] [12], while piRNAs are found only in animals [15]. miRNAs are generated from precursor transcripts by two RNase III-type enzymes, Drosha and Dicer. In animals, single-stranded miRNA is incorporated into the argonaute (Ago) protein complexes (Ago) known as RNA induced silencing complexes (RISC) and binds by partially or completely complementary to the 3′ untranslated region (3′UTR) of a target mRNA [6], this results negative control of gene expression by cleavage or inhibition of translation or other regulatory functions [16]. The biological function of miRNAs was first demonstrated in *C. elegans*, where two miRNAs (*Let*-7 and *Lin*-4) were shown to be regulators for stage-specific differentiation of the worm [17], [18]. Endogenous siRNAs are mainly derived from three sources: transposable elements (TEs), complementary annealed transcripts, also called natural antisense transcripts (NAT) and long ‘fold-back’ transcripts called hairpin RNAs (hpRNAs) [19]. Unlike miRNAs, endo-siRNA's function requires perfect match with the target mRNA [6], [20]. MiRNAs and endogenous siRNAs play important roles in the regulation of fundamental cellular processes, including cell differentiation, stress response, apoptosis, proliferation [12] and metabolism [21], [22]. Recent studies further differentiated the functions of endo-siRNAs based on the sources they were generated from [14]. The TE-derived siRNAs are likely functional in the germline cells by repression of transposon activity and consequently keep genome stability, while NAT-derived siRNAs are more functional in somatic cells [14].

In this study, the transcriptional profiles of both miRNA and endo-siRNA in three developmental stages, namely adult (Ad), new born larvae (NBL) and muscle larvae (ML) of *T. spiralis* were systematically investigated and compared by high throughput cDNA sequencing technology (“RNA-seq”). We found that miRNAs were mainly expressed in the adult worm stage and endo-siRNAs were predominantly derived from transposable elements in the genome.

## Results and Discussion

### Summary of small RNA sequencing

**Figure 1:**
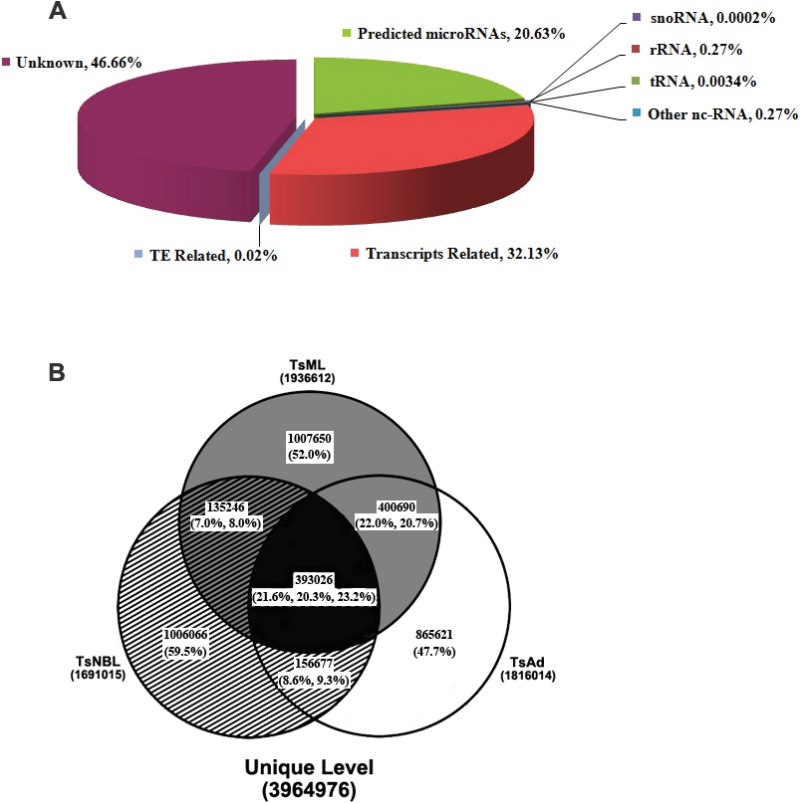
Small non-coding RNA identified in three developmental stages of *T. spiralis*. **A** The composition in percentages of clean reads of small RNAs. **B** Numbers and proportions (bracketed) of unique small RNAs identified in the three stages.

To identify miRNA and endo-siRNA involved in the development of *T. spiralis*, small RNA libraries were generated from three life cycle stages, the new-born larvae, musclar larvae and adult worms. The libraries were directly sequenced using Solexa sequencing technology. A total of about 40 million unfiltered sequence reads (12,368,833 reads from adult worms, 12,867,246 reads from newborn larvae and 14,495,649 reads from muscular larvae) with sizes between 18 and 30 nucleotides were obtained, respectively. After removal of low quality reads and adaptor sequences, the clean reads obtained in Ad, NBL and ML were 11,878,917 (96.0% of total reads), 12,357,960 (96.0% of total reads) and 14,078,375 (97.1% of total reads) respectively. Sequence characterization suggested that the small RNA pools contained miRNAs (20.63%), other non-coding RNAs (rRNA, tRNA, snoRNA) (0.55%), mRNA-related small RNAs (32.13%), TE-related small RNAs (0.02%) and unknown small RNA transcripts (46.66%) (Fig. 1A, Table S1). The GC content of the small RNAs was around 38.7% and the distribution of the small RNA populations in the libraries generally followed the patterns identified in other organisms, such as schistosomal parasites [23]–[29]. The libraries generated in this study have significantly high coverage than those in a recent report, which only showed a proportion of miRNAs in one single stage of the parasite [30].

As shown in Fig. 1B, the unique clean reads obtained in Ad, NBL and ML were 1,816,014, 1,691,015 and 1,936,612, respectively (Table S2). The number of small RNAs existed in all three life cycle stages was 393,026 reads, which accounted for 21.6%, 20.3% and 23.2% of the unique clean reads of Ad, NBL and ML stage, respectively (Fig. 1B). The small RNAs commonly expressed in Ad stage and ML stage were 3 times more than that between Ad stage and NBL stage (Fig. 1B), indicating that many genes of Ad stage were activated already in ML and NBL had more stage-specific small RNAs.

### Length variation of small RNAs in *T. spiralis*

**Figure 2:**
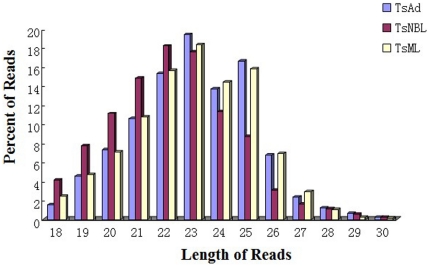
Length distribution of small RNAs in different developmental stages. Length of small RNAs is given on the x-axis in base pairs. And he left Y-axes indicate the percentage of small RNAs afer removal of low quality sequences but prior to selection for complete matches to the genome sequence.

The length of predicted small RNAs varied from 18 to 30 nt in the three life stages, with most of them between from 18 to 27 nt. Here the size and number of sequences in Ad and ML almost followed the same pattern, whereas the number of sequences between 18 and 22 nt was enriched in NBL (Fig. 2 and Table S3). This implied that, apart from stage-specific activation in NBL, small RNAs may undergo size differentiation during early development of the parasite. A previous study indicated that the length of the non-conserved connector helix in Dicer was the main determinant of product size of the mature small RNAs [31], providing a possible explanation for the stage-specific variation we observed. The mechanism behind the stage-specific small RNA processing remains unknown. Since both piRNA and endo-siRNA were more common in the germline, it is likely that the small RNAs found in NBL were predominantly miRNAs, which may play fundamental roles in stage-specific differentiation. Longer RNAs may be more important for parasite reproduction [32]–[37].

### Identification of miRNAs in *T. spriralis*

To identify candidate miRNAs of *T. spiralis*, the clean unique reads obtained were mapped to the draft *T. spiralis* genome sequences (http://genome.wustl.edu/pub/organism/Invertebrates/Trichinella_spiralis/assembly/Trichinella_spiralis-1.0/) [5] sing SOAP. A total of 72,110 (out of 2,099,966) unique small RNA reads that perfectly matched are referred to as miRNA candidates. After screening for secondary structure of the inverted repeats (found with Einverted of Emboss) with RNAfold and evaluation by MirCheck, a total of 240 *T. spiralis* predicted miRNAs were identified.

**Figure 3:**
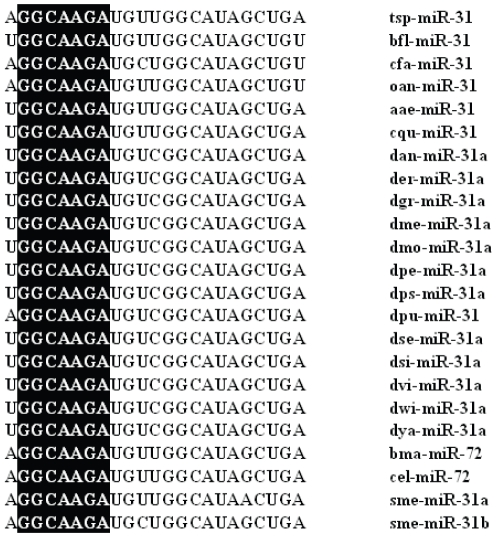
**Alignment of the** ***tsp*** **-mir-31 sequence with homologues from other organisms.** The seed sequences, typically positioned at the 5′ side 2–8 nucleotides, are shadowed in black. Abbreviations: *B. floridae, bfl; C. familiaris, cfa; A.aegypti, aae; O.anatinus, oan; C. quinquefasciatus, cqu; D. grimshawi, dgr; D. ananassae, dan; D. erecta, der; D. melanogaster, dme; D. mojavensis, dmo; D. persimilis, dpe; D. pseudoobscura, dps; D. sechellia, dse; D. simulans, dsi; D. willistoni, dwi; D. virilis, dvi; D. yakuba, dya; B. malayi, bma; D. pulex, dpu; C. elegans, cel; S. mediterranea, sme.*

To further characterize miRNAs in *T. spiralis*, all predicted miRNAs obtained above were compared against a miRNA database, miRBase (Release 15.0). In total, 21 miRNAs were found that had been identified in other species, and belong to 13 different miRNA families (Table 1, Table S4 and S5). Most miRNAs contain a 7 nt region (typically positioned at the 5′ side 2–8 nt) known as the “miRNA seed” sequence (Fig. 3). It has been suggested that the seed regions serv to anchor miRNAs to their mRNA targets [14].

**Table 1:** Conserved (common) miRNAs identified in different developmental stages.

Name	Mature Arm	miR*^a^	Most abundant sequence	Length	Expression (TPM^b^)		
					Ad	NBL	ML
*tsp*-miR-228	5′	Y	AAUGGCACUGGAUGAAUUCACGG	23	23725	19859	60017
*tsp*-miR-100	5′	Y	AACCCGUAGAUCCGAACUUGUGU	23	3028	35995	541
*tsp*-let-7	3′	N	UGAGGUAGUAGGUUGUAUAGUU	22	25573	1067	5554
*tsp*-miR-1	3′	Y	UGGAAUGUAAAGAAGUAUGUAG	22	1515	9759	3351
*tsp*-miR-31	5′	N	AGGCAAGAUGUUGGCAUAGCUGA	23	1004	7728	2025
*tsp*-miR-125	5′	Y	UCCCUGAGACCCAAACUUGUGA	22	745	10	414
*tsp*-miR-252	5′	Y	CUAAGUAGUAGUGCCGCAGGUC	22	193	279	290
*tsp*-miR-9-1	5′	Y	UCUUUGGUUAUCUAGCUGUAUGA	23	239	203	179
*tsp*-miR-87	3′	N	GUGAGCAAAGUUUCAGGUGUGU	22	78	160	120
*tsp*-miR-9-2	3′	Y	AUAAGCUAGUUGACCAAAGA	20	47	25	97
*tsp*-miR-29	3′	Y	UAGCACCAUUUGAAUUCAGUG	21	21	8	24
*tsp*-miR-9-3	3′	Y	UAAAGCUGGAUGACCAAAGU	20	18	8	26
*tsp*-miR-993	3′	Y	GAAGCUCGUUUCUACAGG	18	5	5	16
*tsp*-miR-133	5′	Y	ACUGGUUGAGGACGUACCAAAUUG	24	1	3	1
*tsp*-miR-34	5′	Y	UGGCAGUGUAAUUAGCUGGUUGU	23	1	1	2

a Y indicates that sequences from both arms of a pre-miRNA species were found, while N means that only a sequence from one arm was identified.

b The abundance value of each miRNA was normalized to “transcripts per million (TPM)”. If the value after normalization was less than 1, the normalized value was set as 1.

doi:10.1371/journal.pone.0026448.t001

**Figure 4:**
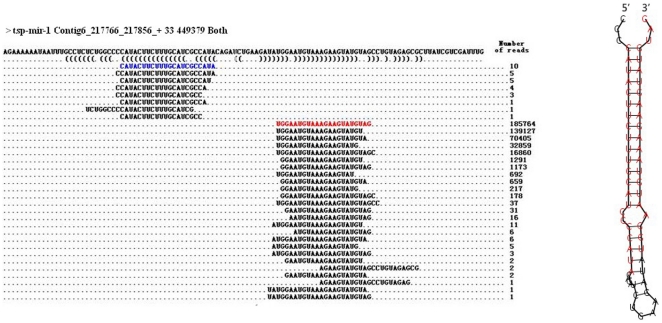
Sequence and predicted secondary stem-loop structure of *tsp*-mir-1 identified in *T. spiralis*. Sequences and the number of sequencing reads matching the *tsp*-mir-1 hairpin were listed. The mature miRNA of *tsp*-mir-1 and the complementary miR* are represented in red and blue respectively. The predicted structure of the pre-miRNA is represented on the right side.

**Figure 5:**
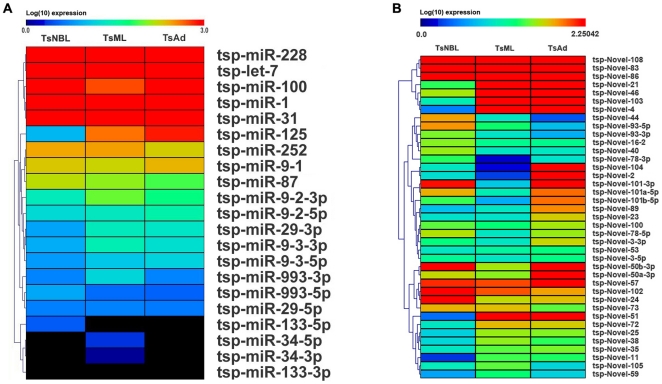
Clustering of expression level of conserved (A) and novel (B) miRNAs. Log _10_ (expression) is used instead of raw expression. The abundance of each miRNA was normalized to “transcripts per million (TPM)”. If the value after normalization was less than 1, the normalized value was set as 1 to avoid the negative values.

The sequencing data showed that, of the 21 conserved miRNAs, *tsp*-let-7 and *tsp*-miR-87 were found to locate only in the 3′ arm of their pre-miRNAs, and *tsp*-miR-31 was located only in the 5′ arm of the hairpin structures. The remaining 18 miRNAs were derived from both arms of the pre-miRNAs (Table S4 and S5). According to the current model of miRNA maturation, Dicer recognizes the double-strand of pre-miRNAs, cleaves away the loop structure and de-associates the duplex to generate antisense and sense strands, *i.e.* the mature miRNA and miRNA* [38]. Although the antisense and sense strands may have different thermostability, which also determined there relative abundance in different tissues, both strands could be functional in miRNA-mediated regulatory pathways [39]. For instance, *tsp*-miR-1-3p was detected at 1,515 TPM (transcripts per million) in the Ad stage, 9,759 in the NBL stage and 3,351 in the ML stage, respectively, whereas its counterpart *tsp*-miR-1-5p was much less abundant (Fig. 4, Table S5). The TPM of *tsp*-miR-100-5p and *tsp*-miR-100-3p, *tsp*-miR-125-5p and *tsp*-miR-125-3p, *tsp*-miR-9-1-5p and *tsp*-miR-9-1-3p, *tsp*-miR-9-2-3p and *tsp*-miR-9-2-5p behaved in similar fashion (Fig. 4, Fig. 5A and Table S5). Thus the anti-sense strands complementary to mRNA targets may play a main regulatory role. However, it cannot be ruled out that both strands generated from the same pre-miRNA execute similar functions. This is because, in some miRNAs, both strands were either similarly expressed or showed stage-specific expression patterns (Fig. 5 A and B; Table S5 and S6).

We found 213 novel miRNAs (Table 2, Table S7). Comparison of novel miRNAs expressed in the three developmental stages of *T. spiralis* revealed stage-specific expression patterns (Fig. 5). A majority of the novel miRNAs in NBL stage has a lower expression level than those in Ad stage and ML stage (Fig. 5B, Table S6 and S7). For instance, *tsp*-novel-21, *tsp*-novel-50a-3p, *tsp*-novel-50b-3p were relatively enriched in adult worm, *tsp*-novel-46 were abundant in muscular larvae, whereas *tsp*-novel-108 and *tsp*-novel-83 exhibited a high abundance in all the three stages (Table S7). Of the novel miRNAs, the expression patterns of *tsp*-novel-93-5p and *tsp*-novel-93-3p, *tsp*-novel-50a-3p and *tsp*-novel-50b-3p, *tsp*-novel-101-3p and *tsp*-novel-101a-5p and *tsp*-novel-101b-5p were interesting. *Tsp*-novel-93-5p and *tsp*-novel-93-3p were derived from the same pre-miRNA, but *tsp*-novel-93-5p was mainly expressed in the NBL stage, less in ML and least in Ad. While *tsp*-novel-93-3p was much less expressed than *tsp*-novel-93-5p in all three stages. (Fig. 5B, Table S7). *Tsp*-novel-50a-3p and *tsp*-novel-50b-3p were derived from the 3′ arm of two gene copies with a similar sequence. *Tsp*-novel-50a-3p was dominantly expressed in Ad, while *tsp*-novel-50b-3p was mainly expressed in NBL and Ad but less in ML. The genes coding for *tsp*-novel-101-3p and *tsp*-novel-101a-5p and *tsp*-novel-101b-5p and their expression patterns were even more complicated. They were encoded by three genes in the genome of *T. spiralis*. The sequences of *tsp*-novel-101-3p derived from the 3 genes were the same, while *tsp*-novel-101b-5p was derived from 1 gene which had a single nucleotide change from *tsp*-novel-101a-5p (Fig. 5B and Table S6 and S7). The later was derived from two gene copies. All these data suggested that there may be multiple layers of control for the stage-specific expression of miRNA genes, as well as their potential regulatory function in the development of the parasite [40]. Although the mRNA targets of these regulatory miRNAs have not been identified, with the availability of the genome sequence of the parasite, genome-wide association study can be pursued. For instance, miR-1 has been found in many species, from *Drosophila* to human [41]–[43], suggesting that they are evolutionary conserved, which has been characterized to play essential functions in regulating proliferation and differentiation of muscle cell [41],[44]. One homologous to mir-1, *tsp*-miR-1, was observed to express in three developmental stages of *T. spiralis* and may have similar functions. Further, MiR-100 and *let*-7, the two conserved miRNA in metazoa, play a role in regulation of developmental timing [18],[42],[45]. Their homologs, *tsp*-miRNA-100 and t*sp*-let-7, were found throughout the life cycle of *T. spiralis*. *Tsp*-let-7 showed very low expression in NBL stage, whereas *tsp*-miRNA-100 was detected in rather high abundance at the same development stage. The abundance of *tsp*-miRNA-100 was almost identical with that of *tsp*-let-7 in both Ad and ML stage, indicating that miRNA-100 may be more functional in NBL stage.

**Table 2:** Novel miRNAs identified in different developmental stages (only the most abundant are shown here).

MicroRNA Name	Mature Arm	miR*^a^	Most abundant sequence	Length	Expression (TPM^b^)		
					Ad	NBL	ML
*tsp*-novel-21	3′	Y	UCACCGGGUAAUAAUUCACAGC	22	4448	24	348
*tsp*-novel-108	5′	Y	CUUGGCACUGUAAGAAUUCACAGA	24	2705	3722	4804
*tsp*-novel-83	3′	Y	UUGAGCAAUUUUGAUCGUAGC	21	2024	1545	1143
*tsp*-novel-50a	3′	Y	UCACCGGAUACUAAAACACGUU	22	1022	47	36
*tsp*-novel-50b	3′	Y	UCACCGGAUACUAAAACACGUGU	23	854	288	40
*tsp*-novel-103	5′	Y	UUUUUAUGAAGUGGUAAGUAGG	22	821	12	623
*tsp*-novel-86	3′	Y	UGAGAUCACCGUGAAAGCCUUU	22	696	333	871
*tsp*-novel-46	3′	Y	UGGACGGCGAAUUAGUGGAAG	21	539	40	1789
*tsp*-novel-4	3′	Y	UGGACGGAUGCUCAGUGGAUGU	22	459	3	860
*tsp*-novel-104	3′	Y	UCACCGGGCACAAUUUGGCUGC	22	270	1	2
*tsp*-novel-2	3′	Y	CACCCGGAUGCUAAAACACGUA	22	194	9	2
*tsp*-novel-51	5′	Y	UCGAAUCGCCACAUCGGAAGGC	22	182	3	171
*tsp*-novel-101	3′	Y	UCACCGGGCACUAAAUCACGUUU	23	178	152	7
*tsp*-novel-57	3′	Y	UUGAGCAAUCACAGUCGUAG	20	170	134	114
*tsp*-novel-89	3′	Y	CAUAGGAUUCUAAAACAUGCA	21	80	6	1
*tsp*-novel-102	5′	Y	ACUGAAAGAGGGAAACGGUUAG	22	69	251	107
*tsp*-novel-24	3′	Y	UGGCAUACUGGAAACGCUGUAGA	23	56	189	46
*tsp*-novel-72	3′	Y	UAAUGAGCAUGUAGACCUGAGU	22	52	1	60
*tsp*-novel-100	3′	Y	GACCAAUGCGUUGAUGUAGA	20	40	29	19
*tsp*-novel-73	5′	Y	UGAAGUUGCACUGGGAUAUGGU	22	29	63	58

a Y indicates that the sequences from both strands of a pre-miRNA species were found, while N means that only the sequence from one arm was identified.

b The abundance value of each miRNA was normalized to “transcripts per million (TPM)”. If the value after normalization was less than 1, the normalized value was set as 1.

doi:10.1371/journal.pone.0026448.t002

### Characterization Of Stage-Associated Mirnas In *T. Spiralis*

**Figure 6:**
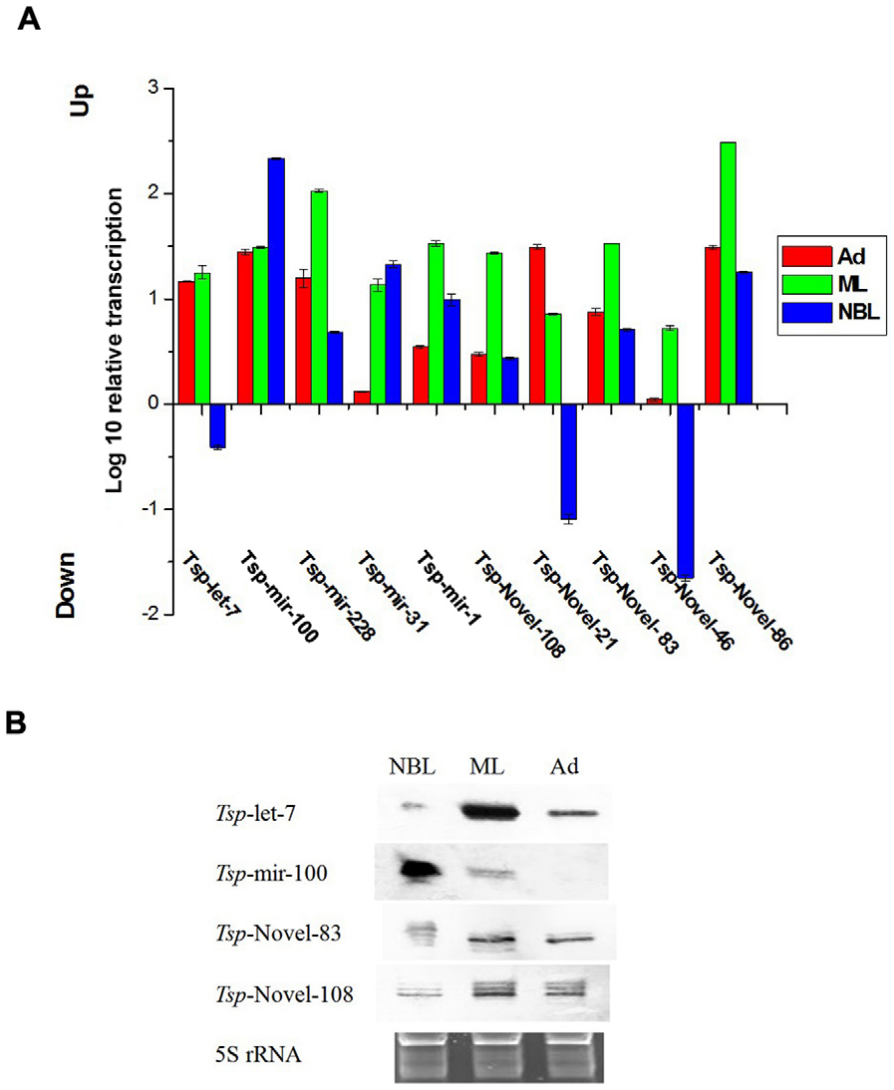
Confirmation of miRNA expression in three developmental stages of *T. spiralis*. **A** The expression level of ten selected miRNAs was in 3 developmental stages of *T. spiralis* measured by qRT-PCR. GAPDH was chosen as an endogenous reference. **B** Identification of the miRNAs by northern-blot. Lanes from left to right: new born larvae (NBL), muscle larvae (ML) and adult (Ad). The 5S rRNA bands were visualized by ethidium bromide staining of polyacrylamide gels and served as loading controls (bottom).

Quantitative real-time PCR and northern blots were performed to validate miRNAs identified in *T. spiralis* and their relative expression levels at different developmental stages. Five conserved miRNAs (*tsp*-miR-228, *tsp*-miR-100, *tsp*-let-7, *tsp*-miR-1 and *tsp*-miR-31) and five novel miRNAs (*tsp*-novel-108, *tsp*-novel-83, *tsp*-novel-46, *tsp*-novel-86 and *tsp*-novel-21) with relatively higher TPM values identified by sequencing were validated by qRT-PCR and Northern blot. First, the above miRNAs identified in sequencing were all amplified by qRT-PCR (Fig. 6A) and except the *tsp*-let-7 which was found less expressed in NBL, the qRT-PCR results were all consistent with the TPM values of sequencing results. The reason for *tsp*-let-7 being found less common in NBL is not known. This may be due to preferential amplification in the tag-generation step before RNA-seq or other unidentified factors. Further, all qPCR amplicons were cloned into the pMD-18T vector (Takara, Dalian, China) and sequenced. The sequencing results demonstrated that the amplicons were identical to the miRNAs sequences (data not shown). Four miRNAs were selected for characterization by northern blots. The results confirmed the qPCR data. However, the probe for *tsp*-mir-100 did not show any signal with total RNA purified from the Ad stage; this was likely due to probe modification or other unidentified factors that prevented annealing of the probes with the targets (Fig. 6B). Since this miRNA was found by both sequencing and qPCR in the Ad stage, we have no doubt that *tsp*-mir-100 was indeed expressed at this stage.

### Identification Of Endogenous Sirna

Endogenous siRNA are extremely diverse and normally not conserved across species. Several types of endogenous siRNAs have been found in *Drosopila melanogaster*, *C. elegans*, *Schistosoma japonicum*, fungi, *Arabidopsis thaliana* and mice [8],[9],[27],[33],[36],[46]–[50]. Most of these endogenous siRNAs derive from transposable elements (TE), complementary annealed transcripts (also called natural antisense transcripts, NAT) and long ‘fold-back’ transcripts called hairpin RNAs (hpRNAs) [19]. TE and NAT are the main sources of endogenous siRNAs. Thus, the small RNA transcript reads perfectly matched to TE and NAT were regarded as endogenous siRNAs (endo-siRNAs) [47],[48].

TEs are major components of the intergenic regions in the genomes of eukaryotic organisms [47],[48]. Based on their structures and modes of integration, TEs are comprised of two main classes [51]. One class includes retrotransposons and retrotransposon-like elements such as Long Interspersed Nuclear Elements (LINE), Long Terminal Repeat Elements (LTR), and Short Interspersed Elements (SINE). The other group includes DNA transposons. We predicted TE structures in the *T. spiralis* genome with RepeatMasker (http://www.repeatmasker.org/), and endo-siRNAs matched to TEs were further analyzed. As reported recently [5], the repetitive sequences in the *T. spiralis* genome estimated to be around 18%, which is much lower than in schistosomes and *Drosophila melanogaster* [52],[53]. Thus, TE-derived siRNAs accounted for a minor portion of the sncRNAs identified here (Fig. 1A).

The numbers for TE-derived siRNAs identified in the Ad, NBL and ML stages were 2,055, 2,234 and 2908 respectively. No obvious stage-related variations in endo-siRNA expression were found, suggesting that TE components in the genome might not be very active during embryogenesis or during other development stages. Further, most of these siRNAs were derived from transposons of Long Interspersed Nucleotide Elements (LINE) family and the transposon Charlie 24 accounted for a major portion (Table S8). Sequencing analysis showed that more siRNA are derived from the antisense strand than from the sense strands (data not shown). This suggested that antisense-derived siRNA may execute regulatory functions through hybridization with the mRNA template generated from the sense strand.

**Figure 7:**
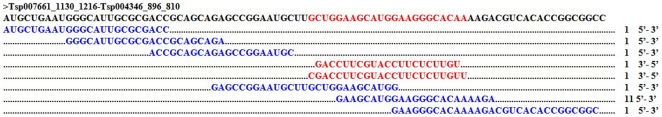
NAT-derived siRNA that matched to the sense and antisense strands. The origin of the genomic sequence (genomic loci) is named on top. siRNAs matched to the sense strand are in blue and those matched to antisense strand are in red. The read numbers and reading direction of the siRNA are listed on the right side.

Since NAT-derived siRNAs were generated from double-stranded RNAs formed by complementary annealed transcripts (NAT) and long ‘fold-back’ transcripts (called hairpin RNAs, hpRNAs) [19], *T. spiralis* NAT-siRNAs were identified from predicted overlapping genes. The number of NAT-derived siRNAs identified in Ad, NBL and ML was 21,157, 16,243 and 21,251, respectively (Table S9). Thus the number of NAT-derived endo-siRNAs was much higher than that from TE, which suggests that these siRNAs played more regulatory roles in the development and parasitization of *T. spiralis*. Further, all NAT-derived siRNAs were trans-NAT siRNAs and no *cis*-NAT siRNAs were found (Fig. 7 and data not shown), which differs from observations with *D. melanogaster* and mice [33],[47]–[49]. This also suggests that there are fewer coding sequences with internal inverted repeat sequences in the genome of *T. spiralis*.

In summary, we identified and analyzed the expression of miRNAs and endo-siRNAs in three development stages of *T. spiralis* through high through-put RNA sequencing techniques. We found vastly more transcripts of miRNAs than that of endo-siRNAs. A total of 21 conserved miRNAs in 13 metazoan miRNA familis and 213 novel miRNAs were identified in the parasite. Some showed clear stage-specific expression patterns, suggesting a potential regulatory function in the corresponding developmental stages. Endo-siRNAs were mainly derived from natural antisense transcripts with TE-derived siRNAs accounting for only minor proportion of the small RNA population. Thus, the function of endo-siRNAs in *T. spiralis* is likely to regulate gene expression instead of maintaining genome stability. The data of this study provide insight information for further dissection of the parasite's biology.

## Materials And Methods

### Parasites

Muscle larvae (ML) of *T. spiralis* (strain ISS534) were obtained from rats 35 days post infection (dpi) by digestion of minced skeletal muscle in 1% pepsin, 1% HCl for 3 h at 37°C with agitation as previously described [54]. To purify adult worms and newborn larvae, Wistar rats at 6 weeks of age were orally inoculated with *T. spiralis* (strain ISS534) with a dose of 8000 larvae per rat. At 30 h or 6 dpi, all rats were killed and the entire intestines were removed, opened longitudinally and cut into small pieces (about 0.5–1 cm). The fragmented intestine was put on a layer of gauze which was immersed into 0.9% sodium chloride solution at 37°C and incubated for 3 h. Adult *T. spiralis* worms migrate into the liquid phase, which was harvested by centrifugation. To obtain new-born larvae, adult worms collected at 6 dpi were incubated in Iscove's Modified Dulbecco's Medium (IMDM) in 75-cm^2^ cell culture plates at 37°C, and the newborn larvae were harvested every 12 h. Our study was reviewed and approved by the Ethics Committee of Jilin University (Ethical clearance application number IZ-2009-III). All animal work was conducted according to Chinese and international guidelines.

### Rna Isolation

Total RNA of *T. spiralis* (Ad, NBL and ML) was extracted using Trizol reagent (Invitrogen, CA, USA) according to the manufacturer's instructions. RNAs were dissolved in diethylpyrocarbonate (DEPC)-treated water, aliquoted and stored at −80°C. RNA was quantified by measuring the absorbance at 260 nm with a Nanodrop 1000 machine (Thermo Scientific CA, USA).

### Construction Of Small Rna Libraries And Sequencing

For small RNA library construction and deep sequencing, the 15–30 nt size range base-pair fraction of each RNA sample from the three life cycle stages was first enriched by 15% TBE urea polyacrylamide gel electrophoresis and the Illumina's proprietary adaptors (UCAGAGUUCUACAGUCCGACGAUC and UCGUAUGCCGUCUUCUGCUUGUidT) were ligated to the 5′ and 3′ termini of the purified small RNAs which were converted into single-stranded cDNA with Superscript II reverse transcriptase (Invitrogen, CA, USA) and Illumina's small RNA RT-Primer. The cDNAs were PCR-amplified with high fidelity Phusion DNApolymerase (Finnzymes Oy, Finland) in 18 PCR cycles using Illumina's small RNA primer sets. After purification using 6% TBE urea PAGE gels, the PCR products were sequenced by Solexa's sequencing-by-synthesis method (Fig. S1).

### Mapping The Sequence Reads Onto The Reference Genome

The bioinformatic analysis and work flow is shown in Figure S1. After removing the low quality sequence reads and the adapter sequences according to the criteria of Illumina, all identical sequences were retained with associated count numbers as their expression abundances To determine whether these clean small RNA sequences were candidate miRNAs, the unique reads were mapped onto the *T. spiralis* genome of the Genome Sequencing Center (GSC) at Washington University St. Louis, (http://genome.wustl.edu/pub/organism/Invertebrates/Trichinella_spiralis/assembly/Trichinella_spiralis-1.0/) with SOAP [55] (http://soap.genomics.org.cn).

The perfectly matched reads were searched against the Metazoa mature miRNA of Sanger miRBase with Patscan [56]. Sequence tags of more than 5 reads that matched perfectly or near-perfectly (no more than 1 mismatch and the mismatch not positioned in the seed region) were regarded to be conserved miRNA candidates. For reads that did not match to the miRNA database, we used the software Einverted of Emboss [57] to find the inverted repeats (stem loops or hairpin structure). Each inverted repeat was extended 10 nt on each side, and the secondary structure of the inverted repeat was predicted by RNAfold [58]. Unique reads with a folding free energy of at least 25 kcal/mole( △*G*°_folding_≤−25 kcal/mol) were evaluated by MirCheck [59] with modified parameters. Finally, precursors (hairpins) of miRNA that passed MirCheck were inspected manually in order to remove false predictions. The reads passing the inspections were regarded as novel miRNAs.

Similar to credibility interval approaches reported for the analysis of SAGE data [60], we employed IDEG6 [61] to identify miRNAs showing statistically significant differences in relative abundance (as reflected by the total count of individual sequence reads) between the three libraries (corresponding to the three developmental stages of the parasite). We used the general Chi-square method for comparison analysis, which has been commonly applied by others [27],[36]. Finally, miRNA with a *P* value≤0.01 were deemed to be significantly different between the samples of the three developmental stages of the parasite.

The repeated sequences, i.e. potential transposable elements, in the *T. spiralis* genome were predicted by using RepeatMasker (http://www.repeatmasker.org/) and the sequences of typical transposons were annotated (data not shown). The sequencing reads that perfectly matched the *T. spiralis* genome were aligned to repeats (TE) using SOAP. The reads that perfectly matched TEs were considered TE-derived siRNAs.

Natural antisense transcripts (NATs) were detected by aligning the *T. spiralis* predicted genes to each other. If a pair of overlapping genes were matched on opposite strands with an E value of ≤1e-9 [62], this pair of overlapping genes was defined as a NAT pair. The reads that perfectly matched the *T. spiralis* genome were aligned to overlapped sequences of NAT pairs with SOAP. The reads that perfectly matched the overlapped regions were considered NAT-derived siRNAs.

### Mirnas Quantification By Real-Time Pcr

Total RNA purified from the three stages were polyadenylated with *E. coli* poly(A) polymerase (E-PAP) following the manufacturer's protocol of the poly(A)-tailing kit (Ambion, CA, USA). The RNA samples were purified separately from the parasites of the same developmental stages for sequencing purpose. After phenol-chloroform extraction and ethanol precipitation, the polyadenylated products were dissolved in DEPC-treated water and reverse-transcribed with 200 U of SuperScript™ III Reverse Transcriptase (Invitrogen, CA) and 3′ RACE Adapter (5′-GCGAGCACAGAATTAATACGACTCACTATAGGT12VN-3′) in the FirstChoice RLM-RACE Kit (Ambion) according to the manufacturer's protocol. The 20 µl RT reaction contained 1 µg total RNA, 2 µl 3′ RACE Adapter, 0.5 mM dNTP mix (Takara, Dalian, China), 10 U RNase inhibitor and 200 U SuperScript™ III Reverse Transcriptase.

In quantitative RT-PCR reactions, GAPDH (glyceraldehyde-3-phosphate dehydrogenase) was chosen as an endogenous reference. The forward and reverse primers for GAPDH were 5′-GTGCTGATTACGCTGTTG-3′ and 5′-CTAAGCCATTGGTAGTGC-3′. PCR was done on an Applied Biosystems 7500 system. The following forward primers were designed to confirm the sequencing results of miRNAs that showed differential expression patterns: *tsp*-miR-100 5′-AAC CCG TAG ATC CGA ACT TGT GT-3′; *tsp*-let-7 5′-TGA GGT AGT AGG TTG TAT AGT T-3′; *tsp*-miR-228 5′-AAT GGC ACT GGA TGA ATT CAC GG-3′; *tsp*-miR-1 5′-TGG AAT GTA AAG AAG TAT GTA G-3′; *tsp*-miR-31 5′-AGG CAA GAT GTT GGC ATA GCT GA-3′; *tsp*-novel-108 5′-CTT GGC ACT GTA AGA ATT CAC AGA-3′; *tsp*-novel-83 5′-TTG AGC AAT TTT GAT CGT AGC-3′; *tsp*-novel-46 5′-TGG ACG GCG AAT TAG TGG AAG-3′; *tsp*-novel-86 5′-TGA GAT CAC CGT GAA AGC CTT T-3′; *tsp*-novel-21 5′-TCA CCG GGT AAT AAT TCA CAG C-3′. The sequence 5′-GCG AGC ACA GAA TTA ATA CGA CT-3′ (complementary to the adaptor) was used as a common reverse primer. Relative expression was calculated by the comparative Ct method [63]. ANOVA and Tukey's HSD post-hoc test were used to analyze significant differences among three stage; *P*<0.05 was considered significant.

### Northern Blot Analysis Of Mirna Expression

Total RNA of *T. spiralis* (muscle larvae, adult worms and newborn larvae) were separated by electrophoresis on a 12.5% polyacrylamide gel under denaturing (8 M urea) conditions and transferred to Hybond-N^+^ nylon membranes (GE Healthsystems, Uppsala, Sweden). The membranes were crossslinked in a UV crosslinker and baked for 1 h at 80°C. Probes complementary to small RNA sequences were end-labeled with DIG at 5′ termini (Takara, Dalian, China). Prehybridization and hybridization were both performed overnight at 53°C in Northernmax Hybridization buffer (Ambion, CA, USA). The blots were washed four times for 30 min in 2×SSC, 0.05% SDS and twice for 15 min in 0.1×SSC, 0.1% SDS at room temperature. The hybridization signal was detected using a DIG Detection Kit (Roche) following manufacturer's instructions. The oligonucleotide probes used for hybridization are as follows:

*tsp*-mir-100 probes: 5′ACACAA*GTTC*GGATCT*AC*GGGTT3′

*tsp*-let-7 probes: 5′AACTAT*ACA*ACCT*ACT*ACCTCA3′

*tsp*-novel-108 probes: 5′TCT*GT*GAATTCTT*ACA*GTGCCAAG3′

*tsp*-novel-83 probes: 5′GCTAC*GATC*AA*AATT*GCTCAA3′

(LNA (Locked nucleic acid) substitutions are indicated by a “*”).

## Supporting Information

Figure S1 **Work-flow of bioinformatics analysis of the small RNA sequences obtained.**
(TIF)

Table S1 General information of the small RNA libraries. (DOC)

Table S2 The common and stage-specific small RNAs in three developmental stages. (DOC)

Table S3 Length distribution of small RNAs in *T.spiralis*. (DOC)

Table S4 Conserved miRNAs identified in different developmental stages. (DOC)

Table S5 Expression levels of conserved miRNAs derived from different arms. (DOC)

Table S6 Novel miRNAs identified in different developmental stages. (DOC)

Table S7 The expression of novel miRNAs derived from different arms. (DOC)

Table S8 siRNAs derived from DNA transposons. (DOC)

Table S9 siRNAs derived from NAT. (DOC)
