# Supplementary figures and images for: Transcriptome of Small Regulatory RNAs in the Development of the Zoonotic Parasite Trichinella spiralis
Source: PLoS One. 2011 Nov 1;6(11):e26448. doi: 10.1371/journal.pone.0026448 (PMC3212509; doi:10.1371/journal.pone.0026448)

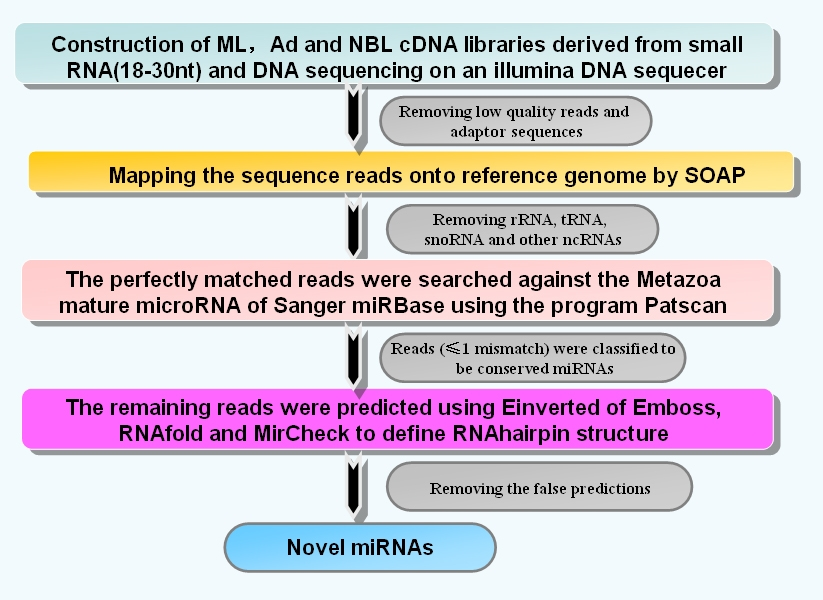

Supplement: Figure S1 — (TIF) [file pone.0026448.s001.tif]
