# Supplementary material for: Transcriptome of Small Regulatory RNAs in the Development of the Zoonotic Parasite Trichinella spiralis
Source: PLoS One. 2011 Nov 1;6(11):e26448. doi: 10.1371/journal.pone.0026448 (PMC3212509; doi:10.1371/journal.pone.0026448)
Supplement: Table S1 — (DOC) [file pone.0026448.s002.doc]

Supplementary Table 1: General information of the small RNA libraries.

| Class | Total | | | | Ad | | | | NBL | | | | ML. | | | |
| --- | --- | --- | --- | --- | --- | --- | --- | --- | --- | --- | --- | --- | --- | --- | --- | --- |
|  | # of Unique | % | Total sequences | % | # of Unique | % | Total sequences | % | # of Unique | % | Total sequences | % | # of Unique | % | Total sequences | % |
| Total Perfect Matched | 2099966 |  | 30008212 |  | 1111407 |  | 9259547 |  | 935327 |  | 9884630 |  | 1126182 |  | 10864035 |  |
| Predicted microRNAs^a^ | 72110 | 3.4 | 6191140 | 20.63 | 38024 | 3.4 | 1777811 | 19.20 | 38135 | 4.1 | 2191442 | 22.17 | 37759 | 3.4 | 2221887 | 20.45 |
| Other Non-coding RNAs^b^ | 4796 | 0.2 | 165287 | 0.55 | 2452 | 0.2 | 75863 | 0.82 | 4234 | 0.5 | 66181 | 0.67 | 2137 | 0.2 | 23243 | 0.21 |
| rRNA | 3881 | 0.2 | 82289 | 0.27 | 2078 | 0.2 | 14320 | 0.15 | 3480 | 0.4 | 57108 | 0.58 | 1820 | 0.2 | 10861 | 0.10 |
| tRNA | 197 | 0.0 | 1034 | 0.00 | 81 | 0.0 | 267 | 0.00 | 141 | 0.0 | 605 | 0.01 | 81 | 0.0 | 162 | 0.00 |
| snoRNA | 14 | 0.0 | 60 | 0.00 | 8 | 0.0 | 9 | 0.00 | 9 | 0.0 | 28 | 0.00 | 7 | 0.0 | 23 | 0.00 |
| other | 704 | 0.0 | 81904 | 0.27 | 285 | 0.0 | 61267 | 0.66 | 604 | 0.1 | 8440 | 0.09 | 229 | 0.0 | 12197 | 0.11 |
| Transcripts Related | 894522 | 42.6 | 9642439 | 32.13 | 464247 | 41.8 | 2814687 | 30.40 | 448488 | 47.9 | 3838685 | 38.83 | 432311 | 38.4 | 2989067 | 27.51 |
| NAT-siRNA | 38121 | 1.8 | 433446 | 1.44 | 21157 | 1.9 | 128170 | 1.38 | 16243 | 1.7 | 144777 | 1.46 | 21251 | 1.9 | 160499 | 1.48 |
| TE Related^c^ | 2090 | 0.1 | 7187 | 0.02 | 829 | 0.1 | 2055 | 0.02 | 1037 | 0.1 | 2234 | 0.02 | 1022 | 0.1 | 2908 | 0.03 |
| LINE | 1 | 0.0 | 1 | 0.00 | 0 | 0.0 | 0 | 0.00 | 1 | 0.0 | 1 | 0.00 | 0 | 0.0 | 0 | 0.00 |
| SINE | 0 | 0.0 | 0 | 0.00 | 0 | 0.0 | 0 | 0.00 | 0 | 0.0 | 0 | 0.00 | 0 | 0.0 | 0 | 0.00 |
| LTR | 82 | 0.0 | 159 | 0.00 | 11 | 0.0 | 24 | 0.00 | 76 | 0.0 | 128 | 0.00 | 6 | 0.0 | 7 | 0.00 |
| DNA | 1626 | 0.1 | 6431 | 0.02 | 795 | 0.1 | 1996 | 0.02 | 639 | 0.1 | 1596 | 0.02 | 971 | 0.1 | 2839 | 0.03 |
| SSR | 80 | 0.0 | 112 | 0.00 | 21 | 0.0 | 33 | 0.00 | 27 | 0.0 | 32 | 0.00 | 40 | 0.0 | 57 | 0.00 |
| Low | 301 | 0.0 | 484 | 0.00 | 2 | 0.0 | 2 | 0.00 | 294 | 0.0 | 477 | 0.00 | 5 | 0.0 | 5 | 0.00 |
| Unknown | 1126448 | 53.6 | 14002159 | 46.66 | 605855 | 54.5 | 4589131 | 49.56 | 443433 | 47.4 | 3786088 | 38.30 | 652953 | 58.0 | 5626930 | 51.79 |
| ^a^ passed miRcheck. | | | |  |  |  | | | | | | | | | | |
| ^b^ Sanger Rfam database release 9.0 ,except microRNA | | | |  |  |  | | | | | | | | | | |
| ^c^ *Trichinella spiralis* Repeats were annotated by Reatmasker | | | | |  |  | | | | | | | | | | |
