# Supplementary material for: Transcriptome of Small Regulatory RNAs in the Development of the Zoonotic Parasite Trichinella spiralis
Source: PLoS One. 2011 Nov 1;6(11):e26448. doi: 10.1371/journal.pone.0026448 (PMC3212509; doi:10.1371/journal.pone.0026448)
Supplement: Table S2 — (DOC) [file pone.0026448.s003.doc]

Supplementary Table 2. The common and stage-specific small RNAs in three developmental stages.

| Comparison | Ad | | | | NBL | | | | ML | | | |
| --- | --- | --- | --- | --- | --- | --- | --- | --- | --- | --- | --- | --- |
|  | Total High-quality Reads | | Unique Clean Reads | | Total High-quality Reads | | Unique Clean Reads | | Total High-quality Reads | | Unique Clean Reads | |
|  | # | % | # | % | # | % | # | % | # | % | # | % |
| All in three stages | 8734439 | 73.5 | 393026 | 21.6 | 8973510 | 72.6 | 393026 | 23.2 | 10776922 | 76.5 | 393026 | 20.3 |
| vs Ad | 1164465 | 9.8 | 865621 | 47.7 | 1184352 | 9.6 | 156677 | 9.3 | 1750127 | 12.4 | 400690 | 20.7 |
| vs NBL | 453515 | 3.8 | 156677 | 8.6 | 1847904 | 15 | 1006066 | 59.5 | 253619 | 1.8 | 135246 | 7 |
| vs Ml | 1526498 | 12.9 | 400690 | 22 | 352194 | 2.8 | 135246 | 8 | 1297707 | 9.2 | 1007650 | 52 |
| In all | 11878917 | 100 | 1816014 | 100 | 12357960 | 100 | 1691015 | 100 | 14078375 | 100 | 1936612 | 100 |
