# Supplementary material for: Transcriptome of Small Regulatory RNAs in the Development of the Zoonotic Parasite Trichinella spiralis
Source: PLoS One. 2011 Nov 1;6(11):e26448. doi: 10.1371/journal.pone.0026448 (PMC3212509; doi:10.1371/journal.pone.0026448)
Supplement: Table S3 — (DOC) [file pone.0026448.s004.doc]

Supplementary Table 3: Length distribution of small RNAs in *T.spiralis.*

| Length | Total Reads | | | | TsAd | | | | TsNBL | | | | TsMl | | | |
| --- | --- | --- | --- | --- | --- | --- | --- | --- | --- | --- | --- | --- | --- | --- | --- | --- |
|  | Unique | | Total | | Unique | | Total | | Unique | | Total | | Unique | | Total | |
|  | # | % | # | % | # | % | # | % | # | % | # | % | # | % | # | % |
| 18 | 96115 | 2.4 | 481748 | 1.3 | 23952 | 1.3 | 76832 | 0.6 | 56876 | 3.4 | 286882 | 2.3 | 40006 | 2.1 | 118034 | 0.8 |
| 19 | 205036 | 5.2 | 1463435 | 3.8 | 77303 | 4.3 | 378842 | 3.2 | 114214 | 6.8 | 717310 | 5.8 | 83225 | 4.3 | 367283 | 2.6 |
| 20 | 318679 | 8 | 2675812 | 7 | 129147 | 7.1 | 721579 | 6.1 | 174280 | 10.3 | 1200241 | 9.7 | 134269 | 6.9 | 753992 | 5.4 |
| 21 | 456686 | 11.5 | 4756976 | 12.4 | 189140 | 10.4 | 1334813 | 11.2 | 243197 | 14.4 | 1911270 | 15.5 | 207092 | 10.7 | 1510893 | 10.7 |
| 22 | 630624 | 15.9 | 8459282 | 22.1 | 280753 | 15.5 | 2730913 | 23 | 312437 | 18.5 | 2765625 | 22.4 | 308950 | 16 | 2962744 | 21 |
| 23 | 726813 | 18.3 | 9436736 | 24.6 | 355612 | 19.6 | 2684018 | 22.6 | 314346 | 18.6 | 2986603 | 24.2 | 367290 | 19 | 3766115 | 26.8 |
| 24 | 593063 | 15 | 4971128 | 13 | 281268 | 15.5 | 1552407 | 13.1 | 223774 | 13.2 | 1320383 | 10.7 | 314622 | 16.2 | 2098338 | 14.9 |
| 25 | 541285 | 13.7 | 4568999 | 11.9 | 298113 | 16.4 | 1911760 | 16.1 | 145493 | 8.6 | 659530 | 5.3 | 296263 | 15.3 | 1997709 | 14.2 |
| 26 | 228198 | 5.8 | 793599 | 2.1 | 109577 | 6 | 289900 | 2.4 | 51861 | 3.1 | 190634 | 1.5 | 116591 | 6 | 313065 | 2.2 |
| 27 | 95705 | 2.4 | 334074 | 0.9 | 37705 | 2.1 | 85152 | 0.7 | 25948 | 1.5 | 143901 | 1.2 | 46204 | 2.4 | 105021 | 0.7 |
| 28 | 45591 | 1.1 | 244962 | 0.6 | 19183 | 1.1 | 66339 | 0.6 | 17256 | 1 | 112285 | 0.9 | 16092 | 0.8 | 66338 | 0.5 |
| 29 | 19192 | 0.5 | 99073 | 0.3 | 10004 | 0.6 | 33434 | 0.3 | 8237 | 0.5 | 50660 | 0.4 | 4156 | 0.2 | 14979 | 0.1 |
| 30 | 7989 | 0.2 | 29428 | 0.1 | 4257 | 0.2 | 12928 | 0.1 | 3096 | 0.2 | 12636 | 0.1 | 1852 | 0.1 | 3864 | 0 |
| In all | 3964976 | 100 | 38315252 | 100 | 1816014 | 100 | 11878917 | 100 | 1691015 | 100 | 12357960 | 100 | 1936612 | 100 | 14078375 | 100 |
