# Supplementary material for: Transcriptome of Small Regulatory RNAs in the Development of the Zoonotic Parasite Trichinella spiralis
Source: PLoS One. 2011 Nov 1;6(11):e26448. doi: 10.1371/journal.pone.0026448 (PMC3212509; doi:10.1371/journal.pone.0026448)
Supplement: Table S4 — (DOC) [file pone.0026448.s005.doc]

Supplementary Table 4. Conserved miRNAs identified in different developmental stages.

| MicroRNA Name | Hairpin | Mature Arm | miR*^a^ | Most abundant sequence | Length | Expression^c^ (TPM^b^) | | |
| --- | --- | --- | --- | --- | --- | --- | --- | --- |
|  |  |  |  |  |  | Ad | NBL | ML |
| tsp-miR-228 | Contig3_1955450_1955632_+ | 5' | Y | AAUGGCACUGGAUGAAUUCACGG | 23 | 23725 | 19859 | 60017 |
| tsp-miR-100 | Contig0_1599445_1599572_+ | 5' | Y | AACCCGUAGAUCCGAACUUGUGU | 23 | 3028 | 35995 | 541 |
| tsp-let-7 | Contig0_1608201_1608359_+ | 3' | N | UGAGGUAGUAGGUUGUAUAGUU | 22 | 25573 | 1067 | 5554 |
| tsp--miR-1 | Contig6_217766_217856_+ | 3' | Y | UGGAAUGUAAAGAAGUAUGUAG | 22 | 1515 | 9759 | 3351 |
| tsp--miR-31 | Contig10_1310189_1310291_+ | 5' | N | AGGCAAGAUGUUGGCAUAGCUGA | 23 | 1004 | 7728 | 2025 |
| tsp--miR-125 | Contig0_1610438_1610518_+ | 5' | Y | UCCCUGAGACCCAAACUUGUGA | 22 | 745 | 10 | 414 |
| tsp--miR-252 | Contig13_1277405_1277493_+ | 5' | Y | CUAAGUAGUAGUGCCGCAGGUC | 22 | 193 | 279 | 290 |
| tsp--miR-9-1 | Contig10_74906_75123_+ | 5' | Y | UCUUUGGUUAUCUAGCUGUAUGA | 23 | 239 | 203 | 179 |
| tsp--miR-87 | Contig0_1506866_1506950_- | 3' | N | GUGAGCAAAGUUUCAGGUGUGU | 22 | 78 | 160 | 120 |
| tsp--miR-9-2 | Contig14_355203_355273_+ | 3' | Y | AUAAGCUAGUUGACCAAAGA | 20 | 47 | 25 | 97 |
| tsp--miR-29 | Contig1_391561_391644_- | 3' | Y | UAGCACCAUUUGAAUUCAGUG | 21 | 21 | 8 | 24 |
| tsp--miR-9-3 | Contig14_354939_355006_+ | 3' | Y | UAAAGCUGGAUGACCAAAGU | 20 | 18 | 8 | 26 |
| tsp--miR-993 | Contig0_8559758_8559948_+ | 3' | Y | GAAGCUCGUUUCUACAGG | 18 | 5 | 5 | 16 |
| tsp--miR-133 | Contig6_225256_225367_+ | 5' | Y | ACUGGUUGAGGACGUACCAAAUUG | 24 | 1 | 3 | 1 |
| tsp--miR-34 | Contig0_10751802_10751996_- | 5' | Y | UGGCAGUGUAAUUAGCUGGUUGU | 23 | 1 | 1 | 2 |

^a^Y indicates that the sequences from both strands of a miRNA* species were found, while N means that only the sequence from one strand of a miRNA* was identified.

^b^The abundance value of each miRNA was normalized to “transcripts per million (TPM)”. If the value after normalization was less than 1, the normalized value was set as 1.

^c^The expression of miRNA was the most abundant sequence of the total counts of unique reads.
