# Supplementary material for: Transcriptome of Small Regulatory RNAs in the Development of the Zoonotic Parasite Trichinella spiralis
Source: PLoS One. 2011 Nov 1;6(11):e26448. doi: 10.1371/journal.pone.0026448 (PMC3212509; doi:10.1371/journal.pone.0026448)
Supplement: Table S5 — (DOC) [file pone.0026448.s006.doc]

Supplementary Table 5. Expression levels of conserved miRNAs derived from different arms.

| MicroRNA Name | Most abundant sequence | Length | Expression^b^ (TPM^a^) | | |
| --- | --- | --- | --- | --- | --- |
|  |  |  | Ad | NBL | ML |
| tsp-miR-228-5p | AAUGGCACUGGAUGAAUUCACGG | 23 | 23725 | 19859 | 60017 |
| tsp-miR-100-5p | AACCCGUAGAUCCGAACUUGUGU | 23 | 3028 | 35995 | 541 |
| let-7-3p | UGAGGUAGUAGGUUGUAUAGUU | 22 | 25573 | 1067 | 5554 |
| tsp-miR-1-3p | UGGAAUGUAAAGAAGUAUGUAG | 22 | 1515 | 9759 | 3351 |
| tsp-miR-31-5p | AGGCAAGAUGUUGGCAUAGCUGA | 23 | 1004 | 7728 | 2025 |
| tsp-miR-125-5p | UCCCUGAGACCCAAACUUGUGA | 22 | 745 | 10 | 414 |
| tsp-miR-252-5p | CUAAGUAGUAGUGCCGCAGGUC | 22 | 193 | 279 | 290 |
| tsp-miR-9-1-5p | UCUUUGGUUAUCUAGCUGUAUGA | 23 | 239 | 203 | 179 |
| tsp-miR-87-3p | GUGAGCAAAGUUUCAGGUGUGU | 22 | 78 | 160 | 120 |
| tsp-miR-9-2-3p | AUAAGCUAGUUGACCAAAGA | 20 | 47 | 25 | 97 |
| tsp-miR-125-3p | AUAGGUUUGGGGUUCAGGAGC | 21 | 75 | 4 | 40 |
| tsp-miR-9-2-5p | UCUUUGGUCAUUUAGCUGUAUG | 22 | 30 | 17 | 23 |
| tsp-miR-29-3p | UAGCACCAUUUGAAUUCAGUG | 21 | 21 | 8 | 24 |
| tsp-miR-9-3-3p | UAAAGCUGGAUGACCAAAGU | 20 | 18 | 8 | 26 |
| tsp-miR-100-3p | CACAAGCUCGUGUCUGGGGUGGA | 23 | 9 | 32 | 5 |
| tsp-miR-9-3-5p | UCUUUGGUUACCUAGCUUUAUGA | 23 | 15 | 7 | 12 |
| tsp-miR-993-3p | GAAGCUCGUUUCUACAGG | 18 | 5 | 5 | 16 |
| tsp-miR-993-5p | GCCCUGUAGAUUCGGGCUUUUGUAG | 25 | 3 | 8 | 4 |
| tsp-miR-29-5p | ACUGAAUUCGGAUGAGUGC | 19 | 5 | 5 | 5 |
| tsp-miR-9-1-3p | AUAAAGCUAGGUUACCAAAG | 20 | 3 | 5 | 6 |
| tsp-miR-252-3p | ACCUGCUCUCUGCUACUUAAGA | 22 | 4 | 2 | 3 |
| tsp-miR-133-5p | ACUGGUUGAGGACGUACCAAAUUG | 24 | 1 | 3 | 1 |
| tsp-miR-34-5p | UGGCAGUGUAAUUAGCUGGUUGU | 23 | 1 | 1 | 2 |
| tsp-miR-133-3p | AUUGGUCCCCUUCAACCAGCU | 21 | 1 | 1 | 0 |
| tsp-miR-34-3p | AACGGCUGAUUGCACUGC | 18 | 1 | 0 | 1 |
| tsp-miR-1-5p | CAUACUUCUUUGCAUCGCCAUA | 22 | 1 | 1 | 1 |
| tsp-miR-228-3p | GUGUUUUCAUCAGUGCUAUAA | 21 | 0 | 1 | 0 |

^a^The abundance value of each miRNA was normalized to “transcripts per million (TPM)”. If the value after normalization was less than 1, the normalized value was set as 1.

^b^The expression of miRNA was the most abundant sequence of the total counts of unique reads.
