# Supplementary material for: Transcriptome of Small Regulatory RNAs in the Development of the Zoonotic Parasite Trichinella spiralis
Source: PLoS One. 2011 Nov 1;6(11):e26448. doi: 10.1371/journal.pone.0026448 (PMC3212509; doi:10.1371/journal.pone.0026448)
Supplement: Table S6 — (DOC) [file pone.0026448.s007.doc]

Supplementary Table 6. Novel miRNAs identified in different developmental stages.

| MicroRNA Name | Hairpin | miR*^a^ | Mature Arm | Most abundant sequence | Length | Expression^c^ (TPM^b^) | | |
| --- | --- | --- | --- | --- | --- | --- | --- | --- |
|  |  |  |  |  |  | Ad | NBL | ML |
| tsp-novel-10 | Contig7_1393132_1393205_- | 3' | N | UGACGGAAAGUGAAUGGC | 18 | 0 | 2.9 | 0 |
| tsp-novel-100 | Contig2296_95_155_+ | 3' | Y | GACCAAUGCGUUGAUGUAGA | 20 | 39.6 | 28.7 | 19.3 |
| tsp-novel-101a-1 | Contig5_1921610_1921704_- | 5' | Y | CGUGAUUUAGGUCCUAGUGGU | 21 | 178 | 151.5 | 6.7 |
| tsp-novel-101a-2 | Contig5_1922046_1922129_- | 5' | Y | CGUGAUUUAGGUCCUAGUGGU | 21 | 178 | 151.5 | 6.7 |
| tsp-novel-101b | Contig5_1922485_1922543_- | 5' | Y | CGUGAUUUAGAUCCUAGUGGU | 23 | 178 | 151.5 | 6.7 |
| tsp-novel-102 | Contig6_1005715_1005827_+ | 5' | Y | ACUGAAAGAGGGAAACGGUUAG | 22 | 68.5 | 251.2 | 107.5 |
| tsp-novel-103 | Contig2_248324_248543_- | 5' | Y | UUUUUAUGAAGUGGUAAGUAGG | 22 | 821.4 | 11.7 | 622.9 |
| tsp-novel-104 | Contig5_1921855_1921923_- | 3' | Y | UCACCGGGCACAAUUUGGCUGC | 22 | 270 | 1 | 1.7 |
| tsp-novel-105 | Contig7_1824028_1824094_+ | 5' | Y | UCGGCGAAUGAUACAUUAAAAAUUG | 25 | 7.9 | 11.2 | 28.8 |
| tsp-novel-106 | Contig0_59037_59085_- | 3' | N | UGACUAGAAGGCCUUUUGACAGCG | 24 | 8.9 | 1 | 11.1 |
| tsp-novel-107 | Contig5_523735_523963_- | 5' | Y | CGGGUAUUCUGUCUUCGACUUG | 22 | 2.1 | 1.1 | 1 |
| tsp-novel-108 | Contig3_1955641_1955724_+ | 5' | Y | CUUGGCACUGUAAGAAUUCACAGA | 24 | 2704.5 | 3721.6 | 4803.5 |
| tsp-novel-109 | Contig5_1920076_1920168_- | 3' | Y | UCAUCGGGAACUAAAUCGCGUA | 22 | 17.8 | 1 | 1 |
| tsp-novel-11 | Contig10_493622_493708_- | 5' | Y | UACGACUGUGAUUGCUCAAUUG | 22 | 22.1 | 1.8 | 25.4 |
| tsp-novel-1-1 | Contig0_1074106_1074199_- | 3' | N | UUGAAGAUCGUGUGGAAGGU | 20 | 1 | 3 | 1 |
| tsp-novel-110 | Contig13_1348839_1349006_- | 5' | N | GGUGGCUGUACAUCGUGGACU | 21 | 1.5 | 5.4 | 1.8 |
| tsp-novel-1-10 | Contig6_1578451_1578544_+ | 3' | N | UUGAAGAUCGUGUGGAAGGU | 20 | 1 | 3 | 1 |
| tsp-novel-111 | Contig13_1349497_1349587_- | 3' | Y | UGAAGGAAGACUUUGAGCAGAUC | 23 | 4.2 | 1 | 9.8 |
| tsp-novel-12 | Contig26_6205_6328_+ | 3' | N | AUAUCUUAGUGAGCGUCAGG | 20 | 1 | 11 | 0 |
| tsp-novel-1-2 | Contig0_8452793_8452886_- | 3' | N | UUGAAGAUCGUGUGGAAGGU | 20 | 1 | 3 | 1 |
| tsp-novel-1-3 | Contig0_8523913_8524006_- | 3' | N | UUGAAGAUCGUGUGGAAGGU | 20 | 1 | 3 | 1 |
| tsp-novel-13-1 | Contig300_1182_1298_+ | 3' | N | GUUCGGUCGUCAGGGAGGG | 19 | 6.1 | 0 | 2.4 |
| tsp-novel-13-2 | Contig3582_273_389_+ | 3' | N | GUUCGGUCGUCAGGGAGGG | 19 | 6.1 | 0 | 2.4 |
| tsp-novel-13-3 | Contig5653_254_372_+ | 3' | N | GUUCGGUCGUCAGGGAGGG | 19 | 6.1 | 0 | 2.4 |
| tsp-novel-14 | Contig5_1442732_1442799_+ | 3' | Y | AAAUAUCUCGUCAUACUGUCGAUU | 24 | 2.3 | 6.6 | 0 |
| tsp-novel-1-4 | Contig1483_1166_1259_+ | 3' | N | UUGAAGAUCGUGUGGAAGGU | 20 | 1 | 3 | 1 |
| tsp-novel-15 | Contig2_960517_960620_+ | 5' | Y | UUGACUGAUAAGAAUGCCUACU | 22 | 6.9 | 1 | 3 |
| tsp-novel-1-5 | Contig15_66122_66215_+ | 3' | N | UUGAAGAUCGUGUGGAAGGU | 20 | 1 | 3 | 1 |
| tsp-novel-1-6 | Contig2144_279_372_- | 3' | N | UUGAAGAUCGUGUGGAAGGU | 20 | 1 | 3 | 1 |
| tsp-novel-16a | Contig0_3045817_3045862_- | 3' | Y | GUGAAGGAUUGUCAGCAAUGUUU | 23 | 0 | 11.2 | 1.9 |
| tsp-novel-16b | Contig5_151773_151818_+ | 3' | Y | UGUGAAGGAUUGUCAGAAAUGUUUG | 25 | 16.2 | 34.6 | 16.1 |
| tsp-novel-17 | Contig3291_208_404_+ | 3' | N | UUUAAGGACGCUGUGAACGC | 20 | 6 | 0 | 3 |
| tsp-novel-1-7 | Contig22_56468_56561_+ | 3' | N | UUGAAGAUCGUGUGGAAGGU | 20 | 1 | 3 | 1 |
| tsp-novel-18 | Contig1_21431_21609_- | 3' | Y | AUAAACUGGCGAACUUGAAG | 20 | 0 | 8.3 | 0 |
| tsp-novel-1-8 | Contig5301_608_701_- | 3' | N | UUGAAGAUCGUGUGGAAGGU | 20 | 1 | 3 | 1 |
| tsp-novel-19 | Contig5_383546_383784_- | 5' | Y | AAGUUUAUAGAUCAGGUUUUGAG | 23 | 1 | 1 | 1 |
| tsp-novel-1-9 | Contig5420_1_92_- | 3' | N | UUGAAGAUCGUGUGGAAGGU | 20 | 1 | 3 | 1 |
| tsp-novel-20 | Contig11_1402431_1402584_- | 3' | Y | CAUGCAGCUCGGCUUGGAUCAGC | 23 | 5.6 | 0 | 0 |
| tsp-novel-21 | Contig0_1673987_1674224_- | 3' | Y | UCACCGGGUAAUAAUUCACAGC | 22 | 4448.1 | 23.7 | 348.3 |
| tsp-novel-2-1 | Contig5_1918112_1918174_- | 3' | Y | CACCCGGAUGCUAAAACACGUA | 22 | 194.4 | 8.8 | 1.9 |
| tsp-novel-22 | Contig5_1950847_1951079_+ | 5' | Y | UGAGGAGAUUCGUCUGGCAUGG | 22 | 4.9 | 6.6 | 7.8 |
| tsp-novel-2-2 | Contig5_1918875_1919111_- | 3' | Y | CACCCGGAUGCUAAAACACGUA | 22 | 194.4 | 8.8 | 1.9 |
| tsp-novel-2-3 | Contig5_1918882_1919113_- | 3' | Y | CACCCGGAUGCUAAAACACGUA | 22 | 194.4 | 8.8 | 1.9 |
| tsp-novel-23-1 | Contig1102_698_790_- | 5' | Y | UUUCUUCGAUUUUGAUUCAAGG | 22 | 7.7 | 1.9 | 5.8 |
| tsp-novel-23-2 | Contig4709_565_657_+ | 5' | Y | UUUCUUCGAUUUUGAUUCAAGG | 22 | 7.7 | 1.9 | 5.8 |
| tsp-novel-23-3 | Contig515_1541_1633_- | 5' | Y | UUUCUUCGAUUUUGAUUCAAGG | 22 | 7.7 | 1.9 | 5.8 |
| tsp-novel-24-1 | Contig15_584704_584794_- | 3' | Y | UGGCAUACUGGAAACGCUGUAGA | 23 | 56 | 189.2 | 46 |
| tsp-novel-24-2 | Contig5_1447859_1447949_+ | 3' | Y | UGGCAUACUGGAAACGCUGUAGA | 23 | 56 | 189.2 | 46 |
| tsp-novel-25-1 | Contig5_142911_143069_+ | 3' | Y | UGACGAGGGAAGUCUGGACAAACAG | 25 | 33.8 | 7.2 | 37.4 |
| tsp-novel-25-2 | Contig560_754_921_+ | 3' | Y | UGACGAGGGAAGUCUGGACAAACAG | 25 | 33.8 | 7.2 | 37.4 |
| tsp-novel-26-1 | Contig6_1116948_1117062_+ | 5' | Y | CUGCAGAUCGUCAAGCUUGGCU | 22 | 1 | 1 | 1.1 |
| tsp-novel-26-2 | Contig6_1095429_1095558_- | 5' | Y | CUGCAGAUCGUCAAGCUUGGCU | 22 | 1 | 1 | 1.1 |
| tsp-novel-27-1 | Contig10_267021_267112_- | 5' | Y | GUCAACGCCGGCUACCUUUGUCAG | 24 | 1 | 1.3 | 1 |
| tsp-novel-27-2 | Contig2837_329_420_- | 5' | Y | GUCAACGCCGGCUACCUUUGUCAG | 24 | 1 | 1.3 | 1 |
| tsp-novel-28-1 | Contig15_584704_584794_+ | 3' | N | UAGGGCUACAGGAGACUCUGAGA | 23 | 4.5 | 3 | 1 |
| tsp-novel-28-2 | Contig5_1447859_1447949_- | 3' | N | UAGGGCUACAGGAGACUCUGAGA | 23 | 4.5 | 3 | 1 |
| tsp-novel-29-1 | Contig10_267145_267210_- | 3' | N | CGGAUGAAGAAAGAGGUC | 18 | 1 | 15.3 | 1 |
| tsp-novel-29-2 | Contig2837_453_518_- | 3' | N | CGGAUGAAGAAAGAGGUC | 18 | 1 | 15.3 | 1 |
| tsp-novel-3 | Contig2_1651329_1651415_+ | 3' | Y | UCACCGGUCCAUUUUAUCUUCU | 22 | 47.6 | 18.3 | 0 |
| tsp-novel-30-1 | Contig4_2258458_2258498_- | 5' | N | UAGGAAGAUCGGUGCUAAUCUG | 22 | 1.9 | 7 | 1.6 |
| tsp-novel-30-2 | Contig4_2704723_2704763_- | 5' | N | UAGGAAGAUCGGUGCUAAUCUG | 22 | 1.9 | 7 | 1.6 |
| tsp-novel-31-1 | Contig0_6876026_6876093_- | 3' | Y | UCCAUGGACCAGACAGAAUGUUC | 23 | 1 | 4.9 | 1 |
| tsp-novel-31-2 | Contig18_376916_377050_- | 3' | Y | UCCAUGGACCAGACAGAAUGUUC | 23 | 1 | 4.9 | 1 |
| tsp-novel-31-3 | Contig18_380303_380437_- | 3' | Y | UCCAUGGACCAGACAGAAUGUUC | 23 | 1 | 4.9 | 1 |
| tsp-novel-31-4 | Contig5_1566610_1566744_+ | 3' | Y | UCCAUGGACCAGACAGAAUGUUC | 23 | 1 | 4.9 | 1 |
| tsp-novel-31-5 | Contig908_465_532_- | 3' | Y | UCCAUGGACCAGACAGAAUGUUC | 23 | 1 | 1.1 | 1 |
| tsp-novel-32a-1 | Contig15_246013_246093_+ | 5' | Y | UGAACAUCUGCUGGUACCAUUCUG | 24 | 1 | 1.1 | 1 |
| tsp-novel-32a-2 | Contig5_1034348_1034428_+ | 5' | Y | UGAACAUCUGCUGGUACCAUUCUG | 24 | 1 | 1.1 | 1 |
| tsp-novel-32b-1 | Contig0_1179720_1179800_+ | 5' | Y | GUGAACAUCUGGUGGUACCAUUCUG | 25 | 6.1 | 2.3 | 1 |
| tsp-novel-32b-2 | Contig2948_180_260_- | 5' | Y | GUGAACAUCUGGUGGUACCAUUCUG | 25 | 6.1 | 2.3 | 1 |
| tsp-novel-33-1 | Contig14_782766_782876_- | 5' | Y | UGUGUAGCGCUUUCAGAAAGGU | 22 | 1.7 | 1 | 2.9 |
| tsp-novel-33-2 | Contig5000_84_194_+ | 5' | Y | UGUGUAGCGCUUUCAGAAAGGU | 22 | 1.7 | 1 | 2.9 |
| tsp-novel-34-1 | Contig0_9280282_9280356_+ | 5' | Y | UGAAGUCUCCGCUGAACUUGGGCAG | 25 | 8.4 | 1.4 | 3.6 |
| tsp-novel-34-2 | Contig0_9281463_9281537_+ | 5' | Y | UGAAGUCUCCGCUGAACUUGGGCAG | 25 | 8.4 | 1.4 | 3.6 |
| tsp-novel-35-1 | Contig0_9043504_9043598_+ | 5' | Y | GUUUCUGUUAGAUUGUACAAAUGGC | 25 | 32.8 | 1 | 28.1 |
| tsp-novel-35-2 | Contig1928_142_236_- | 5' | Y | GUUUCUGUUAGAUUGUACAAAUGGC | 25 | 32.8 | 1 | 28.1 |
| tsp-novel-36-1 | Contig1591_642_704_- | 5' | Y | GCAGCUUUCGAUGUGAUCAUUCC | 23 | 1 | 5.7 | 1 |
| tsp-novel-36-2 | Contig20_214784_214846_- | 5' | Y | GCAGCUUUCGAUGUGAUCAUUCC | 23 | 1 | 5.7 | 1 |
| tsp-novel-37-1 | Contig2570_834_887_- | 3' | Y | AUGGAGUGCUGUUCAAUUUUUCAACA | 26 | 1.1 | 6.6 | 1 |
| tsp-novel-37-2 | Contig3402_550_603_- | 3' | Y | AUGGAGUGCUGUUCAAUUUUUCAACA | 26 | 1.1 | 6.6 | 1 |
| tsp-novel-38 | Contig0_346974_347125_+ | 3' | Y | UGAAUAAUAGGGGCGACAAGG | 21 | 23.7 | 8.1 | 36.1 |
| tsp-novel-39 | Contig0_1834148_1834276_+ | 3' | Y | GUUUCGGAUAUCAGCGUUCAGA | 22 | 3.5 | 1 | 3.9 |
| tsp-novel-4 | Contig9_209054_209145_+ | 3' | Y | UGGACGGAUGCUCAGUGGAUGU | 22 | 458.6 | 3.2 | 860 |
| tsp-novel-40 | Contig0_3779417_3779553_- | 3' | Y | UGGAUAAAUCAGCAAUGUGGA | 21 | 1 | 37.8 | 0 |
| tsp-novel-41-1 | Contig0_9275649_9275880_+ | 3' | Y | UCGAAGAUUGUUGCAGCCAGGC | 22 | 5.6 | 9.2 | 7.2 |
| tsp-novel-41-2 | Contig18_49475_49618_+ | 3' | Y | UCGAAGAUUGUUGCAGCCAGGC | 22 | 5.6 | 9.2 | 7.2 |
| tsp-novel-41-3 | Contig5_357264_357382_- | 3' | Y | UCGAAGAUUGUUGCAGCCAGGC | 22 | 5.6 | 9.2 | 7.2 |
| tsp-novel-41-4 | Contig5222_1246_1364_+ | 3' | Y | UCGAAGAUUGUUGCAGCCAGGC | 22 | 5.6 | 9.2 | 7.2 |
| tsp-novel-41-5 | Contig5464_68_211_+ | 3' | Y | UCGAAGAUUGUUGCAGCCAGGC | 22 | 5.6 | 9.2 | 7.2 |
| tsp-novel-42 | Contig0_7991249_7991443_- | 3' | Y | UUAUUUAAGUCAAAGUUGUCGG | 22 | 5.7 | 1.1 | 6.1 |
| tsp-novel-43a | Contig352_548_606_- | 5' | Y | GUAGCAGCUCUGUACCCGCGUAUU | 24 | 2.9 | 15.2 | 1 |
| tsp-novel-43b-1 | Contig1172_133_195_- | 5' | Y | GUAGCAGCCCUGUACCCGCGUAUU | 24 | 1 | 3.2 | 1.9 |
| tsp-novel-43b-2 | Contig13_381346_381408_- | 5' | Y | GUAGCAGCCCUGUACCCGCGUAUU | 24 | 1 | 3.2 | 1.9 |
| tsp-novel-43b-3 | Contig5_793920_793982_+ | 5' | Y | GUAGCAGCCCUGUACCCGCGUAUU | 24 | 1 | 3.2 | 1.9 |
| tsp-novel-43b-4 | Contig6_76168_76230_+ | 5' | Y | GUAGCAGCCCUGUACCCGCGUAUU | 24 | 1 | 3.2 | 1.9 |
| tsp-novel-44 | Contig11_154644_154797_+ | 5' | Y | AAAAAACACGACUCAGAGA | 19 | 2.3 | 69 | 1 |
| tsp-novel-45 | Contig0_1181216_1181268_+ | 3' | N | ACAUUGUACAAUAAAAAACGG | 21 | 1 | 5.8 | 1 |
| tsp-novel-46 | Contig9_208753_208834_+ | 3' | Y | UGGACGGCGAAUUAGUGGAAG | 21 | 538.8 | 41 | 1788.6 |
| tsp-novel-47 | Contig0_44047_44148_- | 5' | Y | GUGCGUGUGAAGAAGUUCUGG | 21 | 1 | 1 | 1 |
| tsp-novel-48a | Contig8_2077747_2077793_- | 5' | N | GAUGGAUAUUUAGGUGGUAAGG | 22 | 2.1 | 1.2 | 2.2 |
| tsp-novel-48b | Contig31_5954_6000_- | 5' | N | GAUGGAUGUUUGGGUGGUAAG | 21 | 1.8 | 1 | 1 |
| tsp-novel-49 | Contig7_1535757_1535987_+ | 5' | N | UCACGAGGUCGCGUUCUGACA | 21 | 1.1 | 1.8 | 1 |
| tsp-novel-5 | Contig2984_629_764_+ | 5' | Y | AUCGGCUGCGACUAAACGGAAG | 22 | 1.2 | 2.3 | 1.8 |
| tsp-novel-50a | Contig5_1921211_1921304_- | 3' | Y | UCACCGGAUACUAAAACACGUU | 22 | 1021.6 | 47.2 | 35.7 |
| tsp-novel-50b | Contig5_1920851_1920957_- | 3' | Y | UCACCGGAUACUAAAACACGUGU | 23 | 854 | 288.1 | 39.7 |
| tsp-novel-51 | Contig5_24803_24902_- | 5' | Y | UCGAAUCGCCACAUCGGAAGGC | 22 | 181.7 | 2.8 | 171 |
| tsp-novel-52 | Contig3487_358_487_+ | 5' | Y | GCUGGGUUCGUUGGCGAAUG | 20 | 1 | 2.6 | 1 |
| tsp-novel-53 | Contig0_8387105_8387186_- | 3' | Y | UGAUCGCACGUAAGAAUCACUGGC | 24 | 18.5 | 1 | 14.1 |
| tsp-novel-54 | Contig20_180376_180426_+ | 5' | Y | CUGACUUAAUCGCUGUCGCAGAG | 23 | 1 | 0 | 1.3 |
| tsp-novel-55 | Contig10_4419_4479_+ | 3' | N | UGGCGUAUGUACUUGUGGAUCAAA | 24 | 2.1 | 0 | 4.3 |
| tsp-novel-56 | Contig0_4441_4508_- | 5' | Y | GAAGUUCCGGAAUAUUCUGCAGG | 23 | 3.5 | 2.6 | 1.8 |
| tsp-novel-57 | Contig10_493622_493708_+ | 3' | Y | UUGAGCAAUCACAGUCGUAG | 20 | 170 | 133.8 | 114.1 |
| tsp-novel-58 | Contig2954_583_712_- | 5' | Y | UGAAGUGGCUAUAUUGUUUGAU | 22 | 1 | 3.4 | 1 |
| tsp-novel-59 | Contig0_8566691_8566894_+ | 3' | Y | GGCAGAGCUCAGUUGGAAGAGG | 22 | 7.4 | 4.5 | 21 |
| tsp-novel-6 | Contig3484_371_457_- | 5' | Y | UCCGUACCACUAACAGCAAGCGC | 23 | 1 | 1 | 1 |
| tsp-novel-60 | Contig0_10357876_10357965_+ | 5' | Y | UCAAAGACAUCGGCGGAUCUGAU | 23 | 2.4 | 16.9 | 3.2 |
| tsp-novel-61 | Contig5_1566775_1566860_+ | 5' | Y | UCAGACUUCGUCAUUCGCCAGUGGA | 25 | 4 | 1 | 1 |
| tsp-novel-62 | Contig0_8069566_8069612_- | 3' | Y | UUCGUGAUAUUAGUGGACUGU | 21 | 1 | 1 | 1.1 |
| tsp-novel-63 | Contig7_1577109_1577272_- | 5' | Y | UUACGCGGCGGCUUUGAUCCG | 21 | 2 | 9 | 1.4 |
| tsp-novel-64 | Contig10_1703155_1703356_- | 5' | Y | CUGGAUAAUGUUAAAGAUGUACG | 23 | 1.4 | 15.1 | 0 |
| tsp-novel-65 | Contig11_1166376_1166421_- | 3' | N | UCCACUACUCUAGAAAUGCAAGC | 23 | 6 | 0 | 1.6 |
| tsp-novel-66 | Contig0_4631764_4631836_+ | 3' | Y | UCACAACCUCUAUGAGUAAGG | 21 | 3.7 | 1 | 5 |
| tsp-novel-67 | Contig0_2974827_2974963_- | 3' | Y | AGGUUCGACUGGCUGCCAGCAG | 22 | 2.9 | 0 | 5.3 |
| tsp-novel-68 | Contig5_537500_537548_+ | 5' | N | AUUUGAAAUUGAGCUGUGGU | 20 | 1 | 4.2 | 0 |
| tsp-novel-69 | Contig1302_605_775_+ | 3' | Y | UGUCAGUGGAACAAAGAUCAACC | 23 | 9.3 | 1 | 6.5 |
| tsp-novel-7 | Contig245_770_964_- | 5' | Y | GAGGAGUGAUGAAGAAAUAAAGC | 23 | 1 | 1 | 2.3 |
| tsp-novel-70-1 | Contig15_300544_300624_+ | 3' | Y | AAGGGAGCAGAGCUGGGAAUUUC | 22 | 4 | 3.6 | 4.2 |
| tsp-novel-70-2 | Contig5_1766604_1766684_+ | 3' | Y | AAGGGAGCAGAGCUGGGAAUUUC | 23 | 4 | 3.6 | 4.2 |
| tsp-novel-70-3 | Contig5_223669_223749_- | 3' | Y | AAGGGAGCAGAGCUGGGAAUUUC | 23 | 4 | 3.6 | 4.2 |
| tsp-novel-71 | Contig5_462248_462462_- | 5' | N | UUUGCUUGCAGGGACGCUUUACU | 23 | 1.9 | 1 | 1 |
| tsp-novel-72 | Contig4425_38_181_+ | 3' | Y | UAAUGAGCAUGUAGACCUGAGU | 22 | 51.9 | 1 | 59.7 |
| tsp-novel-73 | Contig0_9584700_9584878_- | 5' | Y | UGAAGUUGCACUGGGAUAUGGU | 22 | 28.6 | 62.7 | 57.7 |
| tsp-novel-74 | Contig5_2224569_2224635_+ | 5' | N | AUGAUGGCAAAGUUCGAACGAG | 22 | 1 | 6.8 | 1.2 |
| tsp-novel-75 | Contig5_1368893_1369064_+ | 5' | Y | CAUCAUCGCAUGAUAGUAGGAUAUC | 25 | 3.1 | 1 | 3.1 |
| tsp-novel-76 | Contig0_16077_16265_+ | 5' | Y | GUAGAGGAUUAGGAGCUUUCUG | 22 | 11 | 1 | 15.8 |
| tsp-novel-77 | Contig15_59289_59348_+ | 5' | N | AAUUUGUGGACAAGGAACGUC | 21 | 1 | 3.6 | 1.2 |
| tsp-novel-78 | Contig5_1919348_1919466_- | 5' | Y | CGUGCUUUAGAUUCUGGUAGUU | 22 | 37.7 | 42.8 | 1 |
| tsp-novel-79 | Contig0_2124_2245_+ | 5' | Y | ACUUUUGGUCGAUCAGCGUC | 20 | 1 | 1.1 | 0 |
| tsp-novel-80-1 | Contig10_893273_893423_+ | 5' | Y | UGAUGAUUUAUAACAGACGUAUG | 23 | 3.7 | 7 | 6.1 |
| tsp-novel-80-2 | Contig14_791679_791735_+ | 5' | Y | UGAUGAUUUAUAACAGACGUAUG | 23 | 3.7 | 7 | 6.1 |
| tsp-novel-80-3 | Contig5284_577_633_- | 5' | Y | UGAUGAUUUAUAACAGACGUAUG | 23 | 3.7 | 7 | 6.1 |
| tsp-novel-81 | Contig5381_460_601_+ | 3' | Y | ACGUGCAGAACCUGAGCUGC | 20 | 4.6 | 12.7 | 1.3 |
| tsp-novel-82 | Contig5_1950094_1950221_+ | 3' | Y | GGGAUGAUCGAUCGACUGACG | 21 | 5.5 | 8.2 | 5.5 |
| tsp-novel-83 | Contig10_493381_493467_+ | 3' | Y | UUGAGCAAUUUUGAUCGUAGC | 21 | 2024.3 | 1544.8 | 1142.7 |
| tsp-novel-84 | Contig7_1670604_1670769_+ | 3' | Y | UGUAGGGAUGUUGAGGUGAGCC | 22 | 4.4 | 2.2 | 2.6 |
| tsp-novel-85 | Contig13_1245729_1245931_- | 5' | Y | AUUUCGCGAGGAGCUGCUGACG | 22 | 1 | 0 | 1 |
| tsp-novel-86 | Contig3_830299_830378_- | 3' | Y | UGAGAUCACCGUGAAAGCCUUU | 22 | 695.9 | 332.9 | 871.1 |
| tsp-novel-87 | Contig24_23376_23524_+ | 5' | N | CAGGAUAACAAAGUAGAGAAAC | 22 | 3.2 | 15.8 | 7.5 |
| tsp-novel-88 | Contig4_972211_972321_+ | 5' | Y | GUGUUAACGGGGCGAAGUUCUGA | 23 | 3.2 | 1 | 1 |
| tsp-novel-89 | Contig5_1918526_1918597_- | 3' | Y | CAUAGGAUUCUAAAACAUGCA | 21 | 79.7 | 6 | 1 |
| tsp-novel-8a | Contig2_81633_81695_- | 3' | Y | UGCAUCGCGGGUAUCAGGGCUGU | 23 | 7.7 | 5.6 | 8.7 |
| tsp-novel-8b-1 | Contig13_495606_495664_- | 3' | Y | UGAAUCGCGGGUAUCAGGGCUGUU | 24 | 3.5 | 1 | 5.8 |
| tsp-novel-8b-2 | Contig13_740458_740520_- | 3' | Y | UGAAUCGCGGGUAUCAGGGCUGUU | 24 | 3.5 | 1 | 5.8 |
| tsp-novel-8b-3 | Contig18_248366_248428_+ | 3' | Y | UGAAUCGCGGGUAUCAGGGCUGUU | 24 | 3.5 | 1 | 5.8 |
| tsp-novel-8b-4 | Contig5_1569814_1569890_- | 3' | Y | UGAAUCGCGGGUAUCAGGGCUGUU | 24 | 3.5 | 1 | 5.8 |
| tsp-novel-8c | Contig5_157228_157282_- | 3' | Y | UGAAUCGCAGGUAUCAGGGCUGU | 23 | 3.8 | 6.3 | 4.1 |
| tsp-novel-9 | Contig0_2880087_2880180_+ | 5' | Y | UGCGCCAGCCAAGCAAGAUCGAAGA | 25 | 1 | 1.2 | 2.6 |
| tsp-novel-90 | Contig5_1369120_1369195_+ | 3' | N | UGGGUGUAGUUCGAUUUGUAAG | 22 | 1.1 | 12.5 | 3.3 |
| tsp-novel-91-1 | Contig0_4255500_4255581_+ | 5' | Y | AUGAAGGGUAAACAACUGCUGG | 22 | 2.3 | 12.2 | 3.1 |
| tsp-novel-91-2 | Contig13_353171_353222_+ | 3' | N | AUGAAGGGUAAACAACUGCUGG | 22 | 2.3 | 12.2 | 3.1 |
| tsp-novel-91-3 | Contig5_216069_216120_- | 3' | N | AUGAAGGGUAAACAACUGCUGG | 22 | 2.3 | 12.2 | 3.1 |
| tsp-novel-92 | Contig0_1792340_1792473_- | 5' | Y | UGGUGGACGCUAGACCGGCAGGC | 23 | 4.9 | 1 | 3.7 |
| tsp-novel-93 | Contig6_1005442_1005520_+ | 5' | Y | GCUGAGAAUAGAGAGAUGGGUUGC | 24 | 5.8 | 74.9 | 21 |
| tsp-novel-94 | Contig5_1400894_1400970_- | 5' | Y | AUUUCUCACAAGUUUAUAGAUCAGG | 25 | 1.1 | 1 | 1.9 |
| tsp-novel-95 | Contig5_233424_233501_- | 5' | Y | UGGGUGAAUUCUCUGAUCGACUGGU | 25 | 8.4 | 1 | 1.2 |
| tsp-novel-96 | Contig991_590_653_+ | 5' | Y | GUUGAUCGUCUCGUCACUGUUAAUG | 25 | 5.4 | 1 | 2.7 |
| tsp-novel-97 | Contig5_2133830_2133903_- | 5' | Y | UUAUAGUGGACUGUAUAUGUUAAU | 24 | 0 | 2.1 | 1 |
| tsp-novel-98 | Contig3_3487740_3487974_- | 5' | Y | UGGUAUUGGAGUUUUCUUGAAU | 22 | 1 | 3.8 | 1 |
| tsp-novel-99 | Contig112_3411_3581_- | 5' | Y | GAUCUUCAUUUGCUGACUUCG | 21 | 1 | 0 | 1 |

^a^Y indicates that the sequences from both strands of a miRNA* species were found, while N means that only the sequence from one strand of a miRNA* was identified.

^b^The abundance value of each miRNA was normalized to “transcripts per million (TPM)”. If the value after normalization was less than 1, the normalized value was set as 1.

^c^The expression of miRNA was the most abundant sequence of the total counts of unique reads.
