# Supplementary material for: Transcriptome of Small Regulatory RNAs in the Development of the Zoonotic Parasite Trichinella spiralis
Source: PLoS One. 2011 Nov 1;6(11):e26448. doi: 10.1371/journal.pone.0026448 (PMC3212509; doi:10.1371/journal.pone.0026448)
Supplement: Table S7 — (DOC) [file pone.0026448.s008.doc]

Supplementary Table7. The expression of novel miRNAs derived from different arms.

| MicroRNA Name | Most abundant sequence | Length | Expression^b^ (TPM^a^) | | |
| --- | --- | --- | --- | --- | --- |
|  |  |  | Ad | NBL | ML |
| hsp-Novel-1-3p | UUGAAGAUCGUGUGGAAGGU | 20 | 1.0 | 3.0 | 1.0 |
| hsp-Novel-100-3p | GACCAAUGCGUUGAUGUAGA | 20 | 39.6 | 28.7 | 19.3 |
| hsp-Novel-100-5p | UCUGAUUCCGCUUUGGUAUGC | 21 | 1.0 | 1.0 | 1.0 |
| hsp-Novel-101a-5p | CGUGAUUUAGGUCCUAGUGGU | 21 | 95.4 | 64.0 | 11.8 |
| hsp-Novel-101-3p | UCACCGGGCACUAAAUCACGUUU | 23 | 178.0 | 151.5 | 6.7 |
| hsp-Novel-101b-5p | CGUGAUUUAGAUCCUAGUGGU | 21 | 92.9 | 25.2 | 4.8 |
| hsp-Novel-102-3p | UACCCGUAUCUUUCUUUCAGUAU | 23 | 10.0 | 30.3 | 13.1 |
| hsp-Novel-102-5p | ACUGAAAGAGGGAAACGGUUAG | 22 | 68.5 | 251.2 | 107.5 |
| hsp-Novel-103-3p | CGGGACAUUUGCGCACUUUGUAAG | 24 | 22.2 | 1.0 | 22.4 |
| hsp-Novel-103-5p | UUUUUAUGAAGUGGUAAGUAGG | 22 | 821.4 | 11.7 | 622.9 |
| hsp-Novel-10-3p | UGACGGAAAGUGAAUGGC | 18 | 0.0 | 2.9 | 0.0 |
| hsp-Novel-104-3p | UCACCGGGCACAAUUUGGCUGC | 22 | 270.0 | 1.0 | 1.7 |
| hsp-Novel-104-5p | AGUCAGAUUGCAAUCACGGU | 20 | 1.0 | 5.7 | 1.0 |
| hsp-Novel-105-3p | CGGAUUUUGUUGUUAUUUGCUCAG | 24 | 1.0 | 0.0 | 2.3 |
| hsp-Novel-105-5p | UCGGCGAAUGAUACAUUAAAAAUUG | 25 | 7.9 | 11.2 | 28.8 |
| hsp-Novel-106-3p | UGACUAGAAGGCCUUUUGACAGCG | 24 | 8.9 | 1.0 | 11.1 |
| hsp-Novel-107-3p | GCGAAUUCCAGAAACUUCUGC | 21 | 1.0 | 1.0 | 0.0 |
| hsp-Novel-107-5p | CGGGUAUUCUGUCUUCGACUUG | 22 | 2.1 | 1.1 | 1.0 |
| hsp-Novel-108-3p | GUGGAUUCUUCUGUGUCAAACG | 22 | 2.8 | 59.2 | 3.0 |
| hsp-Novel-108-5p | CUUGGCACUGUAAGAAUUCACAGA | 24 | 2704.5 | 3721.6 | 4803.5 |
| hsp-Novel-109-3p | UCAUCGGGAACUAAAUCGCGUA | 22 | 17.8 | 1.0 | 1.0 |
| hsp-Novel-109-5p | CGGGCUUUAGAUCCUGAUGGU | 21 | 9.2 | 1.0 | 1.0 |
| hsp-Novel-110-5p | GGUGGCUGUACAUCGUGGACU | 21 | 1.5 | 5.4 | 1.8 |
| hsp-Novel-111-3p | UGAAGGAAGACUUUGAGCAGAUC | 23 | 4.2 | 1.0 | 9.8 |
| hsp-Novel-111-5p | UCUUCUGGAUGUCGGCUUGCG | 21 | 1.0 | 0.0 | 1.4 |
| hsp-Novel-11-3p | AUUGGAGCAACCACUGUCGUCGC | 23 | 1.5 | 1.0 | 4.2 |
| hsp-Novel-11-5p | UACGACUGUGAUUGCUCAAUUG | 22 | 22.1 | 1.8 | 25.4 |
| hsp-Novel-12-3p | AUAUCUUAGUGAGCGUCAGG | 20 | 1.0 | 10.4 | 0.0 |
| hsp-Novel-13-3p | GUUCGGUCGUCAGGGAGGG | 19 | 6.1 | 0.0 | 2.4 |
| hsp-Novel-14-3p | AAAUAUCUCGUCAUACUGUCGAUU | 24 | 2.3 | 6.6 | 0.0 |
| hsp-Novel-14-5p | AACUAUGGUCAGCUAUAUCGG | 21 | 1.0 | 1.0 | 0.0 |
| hsp-Novel-15-3p | UGAGCACAACUCAUCAGCGGUA | 22 | 1.0 | 0.0 | 1.0 |
| hsp-Novel-15-5p | UUGACUGAUAAGAAUGCCUACU | 22 | 6.9 | 1.0 | 3.0 |
| hsp-Novel-16a-3p | GUGAAGGAUUGUCAGCAAUGUUU | 23 | 0.0 | 11.2 | 1.9 |
| hsp-Novel-16b-3p | UGUGAAGGAUUGUCAGAAAUGUUUG | 25 | 16.2 | 34.6 | 16.1 |
| hsp-Novel-16-5p | AUUAAUUGUUGACAAUCUUCCUG | 23 | 1.0 | 1.0 | 0.0 |
| hsp-Novel-17-3p | UUUAAGGACGCUGUGAACGC | 20 | 6.0 | 0.0 | 3.0 |
| hsp-Novel-18-3p | AUAAACUGGCGAACUUGAAG | 20 | 0.0 | 8.3 | 0.0 |
| hsp-Novel-18-5p | UGCAGAUUCGCCGGUAUAUUUUC | 23 | 1.0 | 1.0 | 0.0 |
| hsp-Novel-19-3p | GUGAAAAUCUCAUCUUGUAGGCUUC | 25 | 0.0 | 0.0 | 1.0 |
| hsp-Novel-19-5p | AAGUUUAUAGAUCAGGUUUUGAG | 23 | 1.0 | 1.0 | 1.0 |
| hsp-Novel-2-3p | CACCCGGAUGCUAAAACACGUA | 22 | 194.4 | 8.8 | 1.9 |
| hsp-Novel-2-5p | CGUGCUUUAGAUCCCGGUAG | 20 | 5.8 | 17.2 | 0.0 |
| hsp-Novel-20-3p | CAUGCAGCUCGGCUUGGAUCAGC | 23 | 5.6 | 0.0 | 0.0 |
| hsp-Novel-20-5p | UUGAUUCUGGUCGACGACCGC | 21 | 0.0 | 2.8 | 0.0 |
| hsp-Novel-21-3p | UCACCGGGUAAUAAUUCACAGC | 22 | 4448.1 | 23.7 | 348.3 |
| hsp-Novel-21-5p | UGUGAAUUGUUUCCUCGGUCAUU | 23 | 19.1 | 6.3 | 2.5 |
| hsp-Novel-22-3p | GACCUGCCAGAGUUUCUCGACUU | 23 | 0.0 | 0.0 | 1.0 |
| hsp-Novel-22-5p | UGAGGAGAUUCGUCUGGCAUGG | 22 | 4.9 | 6.6 | 7.8 |
| hsp-Novel-23-3p | UUGGCCUUGAAUGAAAGUGACG | 22 | 1.0 | 1.0 | 1.6 |
| hsp-Novel-23-5p | UUUCUUCGAUUUUGAUUCAAGG | 22 | 7.7 | 1.9 | 5.8 |
| hsp-Novel-24-3p | UGGCAUACUGGAAACGCUGUAGA | 23 | 56.0 | 189.2 | 46.0 |
| hsp-Novel-24-5p | UCAGAGUCUCCUGUAGCCCUAGA | 23 | 1.0 | 1.0 | 1.0 |
| hsp-Novel-25-3p | UGACGAGGGAAGUCUGGACAAACAG | 25 | 33.8 | 7.2 | 37.4 |
| hsp-Novel-25-5p | UUAAUUCGUCUUCAGCCUCUCGA | 23 | 1.0 | 1.0 | 1.0 |
| hsp-Novel-26-3p | AGAAAUUAGUGAACUGCAG | 19 | 1.0 | 0.0 | 1.0 |
| hsp-Novel-26-5p | CUGCAGAUCGUCAAGCUUGGCU | 22 | 1.0 | 1.0 | 1.1 |
| hsp-Novel-27-3p | UGACCGAUCUAGCGGCGUAGA | 21 | 1.0 | 1.0 | 1.0 |
| hsp-Novel-27-5p | GUCAACGCCGGCUACCUUUGUCAG | 24 | 1.0 | 1.3 | 1.0 |
| hsp-Novel-28-3p | UAGGGCUACAGGAGACUCUGAGA | 23 | 4.5 | 3.0 | 1.0 |
| hsp-Novel-29-3p | CGGAUGAAGAAAGAGGUC | 18 | 1.0 | 15.3 | 1.0 |
| hsp-Novel-30-5p | UAGGAAGAUCGGUGCUAAUCUG | 22 | 1.9 | 7.0 | 1.6 |
| hsp-Novel-32a-3p | AUGGAUCGGACAGAAUGCUCA | 21 | 1.0 | 0.0 | 1.0 |
| hsp-Novel-32b-3p | AUGGAUCAGACAGAAUGCUCAGA | 23 | 1.0 | 1.0 | 1.0 |
| hsp-Novel-32a-5p | UGAACAUCUGCUGGUACCAUUCUG | 24 | 1.0 | 1.1 | 1.0 |
| hsp-Novel-32c-3p | UCCAUGGACCAGACAGAAUGUUC | 23 | 1.0 | 4.9 | 1.0 |
| hsp-Novel-32b-5p | GUGAACAUCUGGUGGUACCAUUCUG | 25 | 6.1 | 2.3 | 1.0 |
| hsp-Novel-33-3p | UCAGAACGAUAUACACAGCA | 20 | 1.0 | 0.0 | 1.0 |
| hsp-Novel-33-5p | UGUGUAGCGCUUUCAGAAAGGU | 22 | 1.7 | 1.0 | 2.9 |
| hsp-Novel-3-3p | UCACCGGUCCAUUUUAUCUUCU | 22 | 47.6 | 18.3 | 0.0 |
| hsp-Novel-34-3p | AGUUCAUGCAUAGAGGUCG | 19 | 0.0 | 1.0 | 0.0 |
| hsp-Novel-34-5p | UGAAGUCUCCGCUGAACUUGGGCAG | 25 | 8.4 | 1.4 | 3.6 |
| hsp-Novel-35-3p | UUAUGCCAGUAUACAGAAAAUC | 22 | 1.0 | 0.0 | 1.0 |
| hsp-Novel-35-5p | GUUUCUGUUAGAUUGUACAAAUGGC | 25 | 32.8 | 1.0 | 28.1 |
| hsp-Novel-3-5p | AAGAUGAAAUGGAACGGUUAUU | 22 | 17.3 | 15.4 | 0.0 |
| hsp-Novel-36-3p | CGAUGUCACAUCAAGGUUGG | 20 | 1.0 | 2.1 | 1.0 |
| hsp-Novel-36-5p | GCAGCUUUCGAUGUGAUCAUUCC | 23 | 1.0 | 5.7 | 0.9 |
| hsp-Novel-37-3p | AUGGAGUGCUGUUCAAUUUUUCAACA | 26 | 1.1 | 6.6 | 1.0 |
| hsp-Novel-37-5p | UUAAUGAAAAUGAACUGUACUUC | 23 | 1.0 | 1.8 | 1.0 |
| hsp-Novel-38-3p | UGAAUAAUAGGGGCGACAAGG | 21 | 23.7 | 8.1 | 36.1 |
| hsp-Novel-38-5p | AGGCUUGUAGGCGACCUAUU | 20 | 1.0 | 1.0 | 0.0 |
| hsp-Novel-39-3p | GUUUCGGAUAUCAGCGUUCAGA | 22 | 3.5 | 1.0 | 3.9 |
| hsp-Novel-39-5p | AAUUCUGGGUCGCGGAGAGCUGGG | 24 | 1.2 | 0.0 | 1.1 |
| hsp-Novel-40-3p | UGGAUAAAUCAGCAAUGUGGA | 21 | 1.0 | 37.8 | 0.0 |
| hsp-Novel-40-5p | GCACAUUGGACUGAUUUCC | 19 | 1.0 | 1.0 | 0.0 |
| hsp-Novel-41-3p | UCGAAGAUUGUUGCAGCCAGGC | 22 | 5.6 | 9.2 | 7.2 |
| hsp-Novel-41-5p | ACAGCAACUGGAGCAACAUCU | 21 | 1.0 | 4.5 | 1.0 |
| hsp-Novel-42-3p | UUAUUUAAGUCAAAGUUGUCGG | 22 | 5.7 | 1.1 | 6.1 |
| hsp-Novel-42-5p | GAGGAGUGAUGAAGAAAUAAAGCAG | 25 | 1.9 | 0.0 | 6.8 |
| hsp-Novel-43-5p | GUAGCAGCUCUGUACCCGCGUAUU | 24 | 2.9 | 15.2 | 1.0 |
| hsp-Novel-43a-3p | CCGAAUUGCAGGUAUCAGGGCUGUU | 25 | 1.0 | 0.0 | 0.0 |
| hsp-Novel-43b-3p | CGCGGGUAUCAGGGCUGUUUCA | 22 | 1.0 | 1.0 | 1.0 |
| hsp-Novel-4-3p | UGGACGGAUGCUCAGUGGAUGU | 22 | 458.6 | 3.2 | 860.0 |
| hsp-Novel-44-3p | CUCUCAGAGUCGAGUUUUUCG | 21 | 1.0 | 1.0 | 0.0 |
| hsp-Novel-44-5p | AAAAAACACGACUCAGAGA | 19 | 2.3 | 69.0 | 1.0 |
| hsp-Novel-45-3p | ACAUUGUACAAUAAAAAACGG | 21 | 1.0 | 5.8 | 1.0 |
| hsp-Novel-4-5p | CUCCACUGACAUCUGCGCCAAU | 22 | 1.0 | 0.0 | 1.0 |
| hsp-Novel-46-3p | UGGACGGCGAAUUAGUGGAAG | 21 | 538.8 | 40.1 | 1788.6 |
| hsp-Novel-46-5p | UUUCACUAAUCGUCAUCCCAAU | 22 | 1.0 | 0.0 | 1.5 |
| hsp-Novel-47-3p | GCUUGAACUUCUUUGGCACGUGC | 23 | 1.4 | 1.0 | 1.2 |
| hsp-Novel-47-5p | GUGCGUGUGAAGAAGUUCUGG | 21 | 1.0 | 1.0 | 1.0 |
| hsp-Novel-48-5p | GAUGGAUAUUUAGGUGGUAAGG | 22 | 2.1 | 1.2 | 2.2 |
| hsp-Novel-48-5p | GAUGGAUGUUUGGGUGGUAAG | 21 | 1.8 | 1.0 | 1.0 |
| hsp-Novel-49-5p | UCACGAGGUCGCGUUCUGACA | 21 | 1.1 | 1.8 | 1.0 |
| hsp-Novel-50a-3p | UCACCGGAUACUAAAACACGUU | 22 | 1021.6 | 47.2 | 35.7 |
| hsp-Novel-50a-5p | UCGUGUUUUAGGUCCUGGUGG | 21 | 1.0 | 20.1 | 1.0 |
| hsp-Novel-50b-3p | UCACCGGAUACUAAAACACGUGU | 23 | 854.0 | 288.1 | 39.7 |
| hsp-Novel-50b-5p | CGUGUUUUAGAUUCCGGUAGUU | 22 | 3.5 | 3.0 | 1.0 |
| hsp-Novel-51-3p | GUUUCCGAUGGACGAUUCCA | 20 | 1.0 | 0.0 | 1.0 |
| hsp-Novel-51-5p | UCGAAUCGCCACAUCGGAAGGC | 22 | 181.7 | 2.8 | 170.8 |
| hsp-Novel-52-3p | GUCGAUCAGCGUCACGAGC | 19 | 0.0 | 3.6 | 1.0 |
| hsp-Novel-52-5p | GCUGGGUUCGUUGGCGAAUG | 20 | 1.0 | 2.6 | 1.0 |
| hsp-Novel-53-3p | UGAUCGCACGUAAGAAUCACUGGC | 24 | 18.5 | 1.0 | 14.1 |
| hsp-Novel-53-5p | AUAGUCGCUUCUCUACCGCAUAC | 23 | 2.4 | 1.0 | 1.0 |
| hsp-Novel-5-3p | UGGUUGUAUUGAUUUAACGUAUUUG | 25 | 0.0 | 0.0 | 1.0 |
| hsp-Novel-54-3p | UCAGCGACGUCGAUAGGACAG | 21 | 1.0 | 1.0 | 3.4 |
| hsp-Novel-54-5p | CUGACUUAAUCGCUGUCGCAGAG | 23 | 1.0 | 0.0 | 1.3 |
| hsp-Novel-55-3p | UGGCGUAUGUACUUGUGGAUCAAA | 24 | 2.1 | 0.0 | 4.3 |
| hsp-Novel-5-5p | AUCGGCUGCGACUAAACGGAAG | 22 | 1.2 | 2.3 | 1.8 |
| hsp-Novel-56-3p | GCAGAGUAGUCUCUUGAACUUCGUG | 25 | 1.3 | 1.0 | 1.0 |
| hsp-Novel-56-5p | GAAGUUCCGGAAUAUUCUGCAGG | 23 | 3.5 | 2.6 | 1.8 |
| hsp-Novel-57-3p | UUGAGCAAUCACAGUCGUAG | 20 | 170.0 | 133.8 | 114.1 |
| hsp-Novel-57-5p | CGACGACAGUGGUUGCUCCAAUU | 23 | 9.8 | 3.9 | 4.1 |
| hsp-Novel-58-3p | UUCGGACGGUCAGUGAUUUUC | 21 | 1.0 | 1.0 | 0.0 |
| hsp-Novel-58-5p | UGAAGUGGCUAUAUUGUUUGAU | 22 | 1.0 | 3.4 | 1.0 |
| hsp-Novel-59-3p | GGCAGAGCUCAGUUGGAAGAGG | 22 | 7.4 | 4.5 | 20.2 |
| hsp-Novel-59-5p | UUUUCUGGAUGAGUUCGG | 18 | 1.0 | 0.0 | 1.0 |
| hsp-Novel-60-3p | GGUUCAGCCGAUCCUUGAGG | 20 | 0.0 | 1.0 | 0.0 |
| hsp-Novel-60-5p | UCAAAGACAUCGGCGGAUCUGAU | 23 | 2.4 | 16.9 | 3.2 |
| hsp-Novel-61-3p | UGCUGGCGAGUUGGCGGAAGUCU | 23 | 1.0 | 0.0 | 1.1 |
| hsp-Novel-61-5p | UCAGACUUCGUCAUUCGCCAGUGGA | 25 | 4.0 | 1.0 | 0.2 |
| hsp-Novel-62-3p | UUCGUGAUAUUAGUGGACUGU | 21 | 1.0 | 1.0 | 1.1 |
| hsp-Novel-62-5p | GAGGACCAAUAUAACGAGCGAAAUU | 25 | 1.0 | 1.0 | 1.8 |
| hsp-Novel-63-3p | UGGGUCAAUGACUGAACGACAC | 22 | 0.0 | 1.0 | 0.0 |
| hsp-Novel-63-5p | UUACGCGGCGGCUUUGAUCCG | 21 | 2.0 | 9.0 | 1.4 |
| hsp-Novel-6-3p | UGCGCUUCAGGUUGCGGUACUGA | 23 | 1.0 | 1.0 | 1.0 |
| hsp-Novel-64-3p | GCGUAUAAUUUCACACUUGAGUAG | 24 | 0.0 | 1.0 | 0.0 |
| hsp-Novel-64-5p | CUGGAUAAUGUUAAAGAUGUACG | 23 | 1.4 | 15.1 | 0.0 |
| hsp-Novel-65-3p | UCCACUACUCUAGAAAUGCAAGC | 23 | 6.0 | 0.0 | 1.6 |
| hsp-Novel-6-5p | UCCGUACCACUAACAGCAAGCGC | 23 | 1.0 | 1.0 | 1.0 |
| hsp-Novel-66-3p | UCACAACCUCUAUGAGUAAGG | 21 | 3.7 | 1.0 | 5.0 |
| hsp-Novel-66-5p | CUUACUCAUAAGGUUGUCAUGA | 22 | 1.0 | 0.0 | 1.0 |
| hsp-Novel-67-3p | AGGUUCGACUGGCUGCCAGCAG | 22 | 2.9 | 0.0 | 5.3 |
| hsp-Novel-67-5p | AGACUGCUGGCACCAGCUUGC | 21 | 1.0 | 0.0 | 1.0 |
| hsp-Novel-68-5p | AUUUGAAAUUGAGCUGUGGU | 20 | 1.0 | 4.2 | 0.0 |
| hsp-Novel-69-3p | UGUCAGUGGAACAAAGAUCAACC | 23 | 9.3 | 1.0 | 6.5 |
| hsp-Novel-69-5p | UGUGAGAGGAUCUUUGUCAACU | 22 | 1.0 | 0.0 | 1.0 |
| hsp-Novel-70-3p | AAGGGAGCAGAGCUGGGAAUUUC | 23 | 4.0 | 3.6 | 4.2 |
| hsp-Novel-70-5p | GUGCAAUUGCCGGCUGUGCUCC | 22 | 1.0 | 0.0 | 1.0 |
| hsp-Novel-71-5p | UUUGCUUGCAGGGACGCUUUACU | 23 | 1.9 | 1.0 | 1.0 |
| hsp-Novel-72-3p | UAAUGAGCAUGUAGACCUGAGU | 22 | 51.9 | 1.0 | 59.7 |
| hsp-Novel-72-5p | AGGAUUCUUGUCAGCUCUGUCAG | 23 | 1.0 | 0.0 | 1.0 |
| hsp-Novel-73-3p | GUCCGGCGUGGUGCAACAGUUC | 22 | 1.0 | 1.0 | 1.0 |
| hsp-Novel-73-5p | UGAAGUUGCACUGGGAUAUGGU | 22 | 28.6 | 62.7 | 57.7 |
| hsp-Novel-7-3p | UUAUUUAAGUCAAAGUUGUCG | 21 | 1.0 | 1.0 | 1.0 |
| hsp-Novel-74-5p | AUGAUGGCAAAGUUCGAACGAG | 22 | 1.0 | 6.8 | 1.2 |
| hsp-Novel-75-3p | CGGUUUCCUGAUAUUCUGUCUGA | 23 | 1.1 | 1.0 | 1.1 |
| hsp-Novel-75-5p | CAUCAUCGCAUGAUAGUAGGAUAUC | 25 | 3.1 | 1.0 | 3.1 |
| hsp-Novel-7-5p | GAGGAGUGAUGAAGAAAUAAAGC | 23 | 1.0 | 1.0 | 2.3 |
| hsp-Novel-76-3p | UUCAGGACCUCCAGAUGCUUUCAUG | 25 | 1.0 | 0.0 | 1.0 |
| hsp-Novel-76-5p | GUAGAGGAUUAGGAGCUUUCUG | 22 | 10.9 | 1.0 | 15.8 |
| hsp-Novel-77-5p | AAUUUGUGGACAAGGAACGUC | 21 | 1.0 | 3.6 | 1.2 |
| hsp-Novel-78-3p | UCACCGAAUACUAAAGCACGU | 21 | 9.5 | 22.9 | 1.5 |
| hsp-Novel-78-5p | CGUGCUUUAGAUUCUGGUAGUU | 22 | 37.7 | 42.8 | 1.0 |
| hsp-Novel-79-3p | UUUGCUGCCGACAGUAAUG | 19 | 1.0 | 1.0 | 1.0 |
| hsp-Novel-79-5p | ACUUUUGGUCGAUCAGCGUC | 20 | 1.0 | 1.1 | 0.0 |
| hsp-Novel-80-3p | UUUUUGUUAUCUGUCAUCAAUU | 22 | 1.0 | 0.0 | 1.0 |
| hsp-Novel-80-5p | UGAUGAUUUAUAACAGACGUAUG | 23 | 3.7 | 7.0 | 6.1 |
| hsp-Novel-81-3p | ACGUGCAGAACCUGAGCUGC | 20 | 4.6 | 12.7 | 1.3 |
| hsp-Novel-8a-3p | UGCAUCGCGGGUAUCAGGGCUGU | 23 | 7.7 | 5.6 | 8.7 |
| hsp-Novel-81-5p | UCGGGCUGAGGUUUCAAG | 18 | 1.5 | 11.1 | 1.4 |
| hsp-Novel-8a-1-5p | GUAGCAGUCCUGUACCCGCGUAUU | 24 | 0.0 | 1.0 | 1.0 |
| hsp-Novel-8a-2-5p | GUAGCAGCCCUGUACCCGCGUAUU | 24 | 1.0 | 3.2 | 1.9 |
| hsp-Novel-8b-3p | UGAAUCGCGGGUAUCAGGGCUGUU | 24 | 3.5 | 1.0 | 5.8 |
| hsp-Novel-8b-5p | AGCCCUGUACCCGCGUAUUCGG | 22 | 0.0 | 1.0 | 1.0 |
| hsp-Novel-82-3p | GGGAUGAUCGAUCGACUGACG | 21 | 5.5 | 8.2 | 5.5 |
| hsp-Novel-82-5p | CCGUUUCGAAUCGACUCAUCAUGA | 24 | 1.0 | 0.0 | 0.0 |
| hsp-Novel-83-3p | UUGAGCAAUUUUGAUCGUAGC | 21 | 2024.3 | 1544.8 | 1142.7 |
| hsp-Novel-8-3-3p | UGAAUCGCAGGUAUCAGGGCUGU | 23 | 3.8 | 6.3 | 4.1 |
| hsp-Novel-83-5p | CUACGAUCAUCUUUGCUCAAUU | 22 | 52.2 | 9.5 | 9.5 |
| hsp-Novel-8-3-5p | CAGCACUGUACCCGCGCAUUCGG | 23 | 1.0 | 0.0 | 0.0 |
| hsp-Novel-84-3p | UGUAGGGAUGUUGAGGUGAGCC | 22 | 4.4 | 2.2 | 2.6 |
| hsp-Novel-84-5p | UUAUCCUUCAAUGCGCCCUUCG | 22 | 0.0 | 0.0 | 1.0 |
| hsp-Novel-85-3p | AUCGGUCGCUCCAUUGCGGGGGGC | 24 | 1.0 | 0.0 | 1.0 |
| hsp-Novel-85-5p | AUUUCGCGAGGAGCUGCUGACG | 22 | 1.0 | 0.0 | 1.0 |
| hsp-Novel-86-3p | UGAGAUCACCGUGAAAGCCUUU | 22 | 695.9 | 332.9 | 871.1 |
| hsp-Novel-86-5p | AGGUUUUCACCCUGAUCUAACA | 22 | 1.0 | 1.4 | 1.0 |
| hsp-Novel-87-5p | CAGGAUAACAAAGUAGAGAAAC | 22 | 3.2 | 15.8 | 7.5 |
| hsp-Novel-88-3p | AUUCCUAACAGCUCUGUGUUAAU | 23 | 1.0 | 1.0 | 0.0 |
| hsp-Novel-88-5p | GUGUUAACGGGGCGAAGUUCUGA | 23 | 3.2 | 1.0 | 1.0 |
| hsp-Novel-89-3p | CAUAGGAUUCUAAAACAUGCA | 21 | 79.7 | 6.0 | 1.0 |
| hsp-Novel-89-5p | UCGUGCUUUAGAAUCCGGUA | 20 | 1.0 | 1.0 | 0.0 |
| hsp-Novel-90-3p | UGGGUGUAGUUCGAUUUGUAAG | 22 | 1.1 | 12.5 | 3.3 |
| hsp-Novel-91a-3p | GCAGUUACUUUGAGCCAGCAG | 21 | 1.0 | 1.0 | 1.0 |
| hsp-Novel-91b-3p | AUGAAGGGUAAACAACUGCUGG | 22 | 2.3 | 12.2 | 3.1 |
| hsp-Novel-92-3p | CACCCGGUCGAGAUGGACAUCACAG | 25 | 1.0 | 1.0 | 1.0 |
| hsp-Novel-92-5p | UGGUGGACGCUAGACCGGCAGGC | 23 | 4.9 | 1.0 | 3.7 |
| hsp-Novel-93-3p | UACCCGUACUCUAAUCUUAG | 20 | 5.0 | 32.9 | 9.9 |
| hsp-Novel-93-5p | GCUGAGAAUAGAGAGAUGGGUUGC | 24 | 5.8 | 74.9 | 21.0 |
| hsp-Novel-9-3p | UUCGAGGUCUUCUCUCCGGUGUACU | 25 | 1.0 | 1.0 | 1.0 |
| hsp-Novel-94-3p | UCAUCUAGUAGGCUUGUGAGGU | 22 | 1.0 | 1.0 | 1.1 |
| hsp-Novel-94-5p | AUUUCUCACAAGUUUAUAGAUCAGG | 25 | 1.1 | 1.0 | 1.9 |
| hsp-Novel-95-3p | GUUGUAUCAGGGACUCACCAUG | 22 | 1.6 | 4.4 | 1.0 |
| hsp-Novel-95-5p | UGGGUGAAUUCUCUGAUCGACUGGU | 25 | 8.4 | 1.0 | 1.2 |
| hsp-Novel-9-5p | UGCGCCAGCCAAGCAAGAUCGAAGA | 25 | 1.0 | 1.2 | 2.6 |
| hsp-Novel-96-3p | UUGAAGAGCGUUCAACGG | 18 | 1.0 | 1.0 | 4.4 |
| hsp-Novel-96-5p | GUUGAUCGUCUCGUCACUGUUAAUG | 25 | 5.4 | 1.0 | 2.7 |
| hsp-Novel-97-3p | UUAUUAAUGACAGCGGGUGUGA | 22 | 0.0 | 1.0 | 1.0 |
| hsp-Novel-97-5p | UUAUAGUGGACUGUAUAUGUUAAU | 24 | 0.0 | 2.1 | 1.0 |
| hsp-Novel-98-3p | UAUUACUGAUAACUCUGGUGCU | 22 | 0.0 | 1.0 | 0.0 |
| hsp-Novel-98-5p | UGGUAUUGGAGUUUUCUUGAAU | 22 | 1.0 | 3.8 | 1.0 |
| hsp-Novel-99-3p | GUAGAGUGACAACAAUGAAGAUA | 23 | 1.0 | 1.0 | 7.1 |
| hsp-Novel-99-5p | GAUCUUCAUUUGCUGACUUCG | 21 | 1.0 | 0.0 | 1.0 |

^a^The abundance value of each miRNA was normalized to “transcripts per million (TPM)”. If the value after normalization was less than 1, the normalized value was set as 1.

^b^The expression of miRNA was the most abundant sequence of the total counts of unique reads.
