# Supplementary material for: Transcriptome of Small Regulatory RNAs in the Development of the Zoonotic Parasite Trichinella spiralis
Source: PLoS One. 2011 Nov 1;6(11):e26448. doi: 10.1371/journal.pone.0026448 (PMC3212509; doi:10.1371/journal.pone.0026448)
Supplement: Table S8 — (DOC) [file pone.0026448.s009.doc]

**Supplementary Table 8. siRNAs derived from DNA transposons.**

| TE | TE Class | TE Family | Length | Total | | Sense | | Antisense | |
| --- | --- | --- | --- | --- | --- | --- | --- | --- | --- |
|  |  |  |  | # of Unique | Total Counts | # of Unique | Total Counts | # of Unique | Total Counts |
| Contig2358_88_182_+ | DNA transposons | Charlie14a | 95 | 1 | 1 | 0 | 0 | 1 | 1 |
| Contig2204_972_1066_+ | DNA transposons | Charlie14a | 95 | 1 | 1 | 0 | 0 | 1 | 1 |
| Contig1676_1229_1330_+ | DNA transposons | Charlie24 | 102 | 3 | 4 | 2 | 2 | 1 | 2 |
| Contig445_1620_1721_- | DNA transposons | Charlie24 | 102 | 3 | 4 | 2 | 2 | 1 | 2 |
| Contig6221_602_679_- | DNA transposons | Charlie24 | 78 | 14 | 26 | 4 | 5 | 10 | 21 |
| Contig5773_707_796_+ | DNA transposons | Charlie24 | 90 | 21 | 39 | 3 | 3 | 18 | 36 |
| Contig3731_1018_1095_+ | DNA transposons | Charlie24 | 78 | 29 | 127 | 3 | 3 | 26 | 124 |
| Contig6038_716_793_- | DNA transposons | Charlie24 | 78 | 29 | 127 | 3 | 3 | 26 | 124 |
| Contig1692_937_1129_- | DNA transposons | Charlie6 | 193 | 80 | 294 | 5 | 5 | 75 | 289 |
| Contig528_811_936_- | DNA transposons | Charlie24 | 126 | 80 | 508 | 8 | 13 | 72 | 495 |
| Contig1292_173_427_- | DNA transposons | Charlie6 | 255 | 82 | 296 | 5 | 5 | 77 | 291 |
| Contig6221_189_370_- | DNA transposons | Charlie24 | 182 | 133 | 334 | 26 | 38 | 107 | 296 |
| Contig5773_980_1179_+ | DNA transposons | Charlie24 | 200 | 152 | 498 | 19 | 24 | 133 | 474 |
| Contig1112_2787_2912_+ | DNA transposons | Charlie24 | 126 | 174 | 650 | 19 | 29 | 155 | 621 |
| Contig3699_167_292_+ | DNA transposons | Charlie24 | 126 | 174 | 650 | 19 | 29 | 155 | 621 |
| Contig5420_157_282_+ | DNA transposons | Charlie24 | 126 | 174 | 650 | 19 | 29 | 155 | 621 |
| Contig602_1313_1438_- | DNA transposons | Charlie24 | 126 | 174 | 650 | 19 | 29 | 155 | 621 |
| Contig2045_767_890_+ | DNA transposons | Charlie24 | 124 | 175 | 849 | 19 | 29 | 156 | 820 |
| Contig5301_765_890_+ | DNA transposons | Charlie24 | 126 | 186 | 867 | 19 | 29 | 167 | 838 |
| Contig949_148_273_+ | DNA transposons | Charlie24 | 126 | 194 | 882 | 19 | 29 | 175 | 853 |
| Contig2144_436_561_+ | DNA transposons | Charlie24 | 126 | 194 | 882 | 19 | 29 | 175 | 853 |
| Contig1483_977_1102_- | DNA transposons | Charlie24 | 126 | 194 | 882 | 19 | 29 | 175 | 853 |
| Contig1516_920_1045_+ | DNA transposons | Charlie24 | 126 | 194 | 882 | 19 | 29 | 175 | 853 |
| Contig1490_339_464_+ | DNA transposons | Charlie24 | 126 | 194 | 882 | 19 | 29 | 175 | 853 |
| Contig4388_515_751_+ | DNA transposons | Charlie24 | 237 | 254 | 809 | 21 | 29 | 233 | 780 |
| Contig575_380_560_+ | DNA transposons | Charlie24 | 181 | 258 | 1103 | 19 | 29 | 239 | 1074 |
| Contig1404_43_549_+ | DNA transposons | Charlie24 | 507 | 341 | 857 | 152 | 315 | 189 | 542 |
| Contig2045_43_428_+ | DNA transposons | Charlie9 | 386 | 361 | 1432 | 130 | 261 | 231 | 1171 |
| Contig5301_3_309_+ | DNA transposons | Charlie9 | 307 | 398 | 1334 | 121 | 283 | 277 | 1051 |
| Contig5069_476_980_+ | DNA transposons | Charlie24 | 505 | 502 | 2239 | 102 | 155 | 400 | 2084 |
| Contig1516_20_582_+ | DNA transposons | Charlie9 | 563 | 585 | 2343 | 210 | 468 | 375 | 1875 |
| Contig6038_16_522_- | DNA transposons | Charlie24 | 507 | 690 | 3486 | 150 | 274 | 540 | 3212 |
| Contig5185_222_815_+ | DNA transposons | Charlie24 | 594 | 787 | 3199 | 180 | 377 | 607 | 2822 |
