# Supplementary material for: Transcriptome of Small Regulatory RNAs in the Development of the Zoonotic Parasite Trichinella spiralis
Source: PLoS One. 2011 Nov 1;6(11):e26448. doi: 10.1371/journal.pone.0026448 (PMC3212509; doi:10.1371/journal.pone.0026448)
Supplement: Table S9 — (DOC) [file pone.0026448.s010.doc]

**Supplementary Table 9. siRNAs derived from NAT.**

| NAT | Class | Total | | Sense | | AntiSense | |
| --- | --- | --- | --- | --- | --- | --- | --- |
|  |  | # of Unique | # of Total | # of Unique | # of Total | # of Unique | # of Total |
| Tsp003595_4_783-Tsp007973_4976_4201 | Trans-NAT | 1968 | 28861 | 1206 | 22046 | 762 | 6815 |
| Tsp003595_208_774-Tsp009169_567_1 | Trans-NAT | 1079 | 22057 | 951 | 20126 | 128 | 1931 |
| Tsp008319_74_378-Tsp009464_325_21 | Trans-NAT | 1011 | 9413 | 533 | 4255 | 478 | 5158 |
| Tsp008361_1_287-Tsp009464_287_1 | Trans-NAT | 979 | 8747 | 594 | 4292 | 385 | 4455 |
| Tsp009676_1_458-Tsp006566_1244_782 | Trans-NAT | 902 | 5319 | 598 | 4584 | 304 | 735 |
| Tsp009676_41_458-Tsp007202_1958_1541 | Trans-NAT | 894 | 5318 | 580 | 4551 | 314 | 767 |
| Tsp008857_74_366-Tsp009464_325_30 | Trans-NAT | 889 | 6672 | 413 | 1516 | 476 | 5156 |
| Tsp004855_434_777-Tsp009336_486_143 | Trans-NAT | 874 | 8895 | 374 | 6341 | 500 | 2554 |
| Tsp009676_1_458-Tsp009158_1259_797 | Trans-NAT | 874 | 5198 | 598 | 4584 | 276 | 614 |
| Tsp009336_172_486-Tsp007448_862_548 | Trans-NAT | 843 | 8973 | 494 | 2547 | 349 | 6426 |
| Tsp009676_1_458-Tsp006565_1370_908 | Trans-NAT | 838 | 5125 | 598 | 4584 | 240 | 541 |
| Tsp009354_261_432-Tsp009594_389_218 | Trans-NAT | 809 | 10680 | 524 | 7573 | 285 | 3107 |
| Tsp009670_115_286-Tsp009594_389_218 | Trans-NAT | 809 | 10680 | 524 | 7573 | 285 | 3107 |
| Tsp009996_96_267-Tsp009594_389_218 | Trans-NAT | 809 | 10680 | 524 | 7573 | 285 | 3107 |
| Tsp009336_172_486-Tsp008220_862_548 | Trans-NAT | 803 | 8915 | 494 | 2547 | 309 | 6368 |
| Tsp009304_96_304-Tsp007220_623_410 | Trans-NAT | 793 | 31056 | 288 | 1323 | 505 | 29733 |
| Tsp004415_1_180-Tsp008361_287_108 | Trans-NAT | 792 | 9632 | 345 | 5807 | 447 | 3825 |
| Tsp004415_1_180-Tsp008319_398_219 | Trans-NAT | 750 | 9769 | 345 | 5807 | 405 | 3962 |
| Tsp005183_2304_2787-Tsp008570_747_265 | Trans-NAT | 695 | 5011 | 255 | 910 | 440 | 4101 |
| Tsp004415_1_180-Tsp008093_305_126 | Trans-NAT | 688 | 9355 | 345 | 5807 | 343 | 3548 |
| Tsp009441_4_185-Tsp004285_326_145 | Trans-NAT | 625 | 13173 | 117 | 736 | 508 | 12437 |
| Tsp009611_71_232-Tsp005117_672_512 | Trans-NAT | 625 | 5410 | 340 | 3905 | 285 | 1505 |
| Tsp009441_4_189-Tsp008361_302_117 | Trans-NAT | 571 | 4594 | 119 | 738 | 452 | 3856 |
| Tsp009926_1_351-Tsp004889_1376_1029 | Trans-NAT | 540 | 2766 | 171 | 1302 | 369 | 1464 |
| Tsp009441_4_185-Tsp004251_237_56 | Trans-NAT | 532 | 4437 | 117 | 736 | 415 | 3701 |
| Tsp009979_15_357-Tsp007623_1115_773 | Trans-NAT | 531 | 7351 | 488 | 7299 | 43 | 52 |
| Tsp009979_15_357-Tsp007888_866_524 | Trans-NAT | 531 | 7351 | 488 | 7299 | 43 | 52 |
| Tsp009979_15_357-Tsp008323_2001_1659 | Trans-NAT | 505 | 7323 | 488 | 7299 | 17 | 24 |
| Tsp009979_33_357-Tsp005213_1097_773 | Trans-NAT | 477 | 6969 | 427 | 6909 | 50 | 60 |
| Tsp008914_2_162-Tsp003106_1250_1090 | Trans-NAT | 474 | 12714 | 252 | 11815 | 222 | 899 |
| Tsp009037_53_223-Tsp009549_348_179 | Trans-NAT | 474 | 4164 | 73 | 766 | 401 | 3398 |
| Tsp009441_1_178-Tsp004496_185_8 | Trans-NAT | 451 | 8966 | 114 | 732 | 337 | 8234 |
| Tsp009963_172_469-Tsp008238_3948_3651 | Trans-NAT | 432 | 3108 | 235 | 2544 | 197 | 564 |
| Tsp009963_172_469-Tsp009312_440_143 | Trans-NAT | 432 | 3108 | 235 | 2544 | 197 | 564 |
| Tsp009963_172_469-Tsp009658_471_174 | Trans-NAT | 432 | 3108 | 235 | 2544 | 197 | 564 |
| Tsp000011_16_204-Tsp008032_365_177 | Trans-NAT | 410 | 3974 | 239 | 3520 | 171 | 454 |
| Tsp009963_172_469-Tsp007371_3807_3510 | Trans-NAT | 409 | 3036 | 235 | 2544 | 174 | 492 |
| Tsp009135_2_149-Tsp003106_1250_1103 | Trans-NAT | 406 | 10418 | 186 | 9525 | 220 | 893 |
| Tsp008914_1_176-Tsp008196_6093_5918 | Trans-NAT | 390 | 12210 | 266 | 11844 | 124 | 366 |
| Tsp005347_357_441-Tsp009636_92_8 | Trans-NAT | 379 | 11905 | 233 | 10019 | 146 | 1886 |
| Tsp006654_174_276-Tsp007028_187_85 | Trans-NAT | 373 | 2990 | 200 | 1242 | 173 | 1748 |
| Tsp005114_553_679-Tsp005117_672_546 | Trans-NAT | 372 | 3772 | 158 | 2683 | 214 | 1089 |
| Tsp008914_1_155-Tsp001593_768_614 | Trans-NAT | 366 | 12109 | 250 | 11813 | 116 | 296 |
| Tsp010162_3_564-Tsp005815_2159_1595 | Trans-NAT | 337 | 1847 | 289 | 1782 | 48 | 65 |
| Tsp005012_163_255-Tsp004996_3337_3245 | Trans-NAT | 334 | 8094 | 74 | 189 | 260 | 7905 |
| Tsp005478_541_661-Tsp005481_231_111 | Trans-NAT | 334 | 2880 | 294 | 2774 | 40 | 106 |
| Tsp009596_8_120-Tsp004966_716_604 | Trans-NAT | 308 | 4965 | 194 | 4104 | 114 | 861 |
| Tsp009596_8_120-Tsp004967_140_28 | Trans-NAT | 308 | 4965 | 194 | 4104 | 114 | 861 |
| Tsp009596_8_120-Tsp008585_557_445 | Trans-NAT | 308 | 4965 | 194 | 4104 | 114 | 861 |
| Tsp009135_1_149-Tsp008196_6093_5945 | Trans-NAT | 303 | 9867 | 186 | 9525 | 117 | 342 |
| Tsp005014_54_224-Tsp009230_263_92 | Trans-NAT | 299 | 3230 | 255 | 3139 | 44 | 91 |
| Tsp006522_19_216-Tsp007028_285_85 | Trans-NAT | 299 | 3374 | 41 | 70 | 258 | 3304 |
| Tsp005183_2517_2787-Tsp008552_400_130 | Trans-NAT | 298 | 1334 | 162 | 657 | 136 | 677 |
| Tsp009336_1_143-Tsp007382_1079_937 | Trans-NAT | 295 | 1879 | 226 | 1740 | 69 | 139 |
| Tsp005066_389_507-Tsp008937_119_1 | Trans-NAT | 291 | 1616 | 41 | 85 | 250 | 1531 |
| Tsp009135_1_149-Tsp001593_768_620 | Trans-NAT | 290 | 9792 | 186 | 9525 | 104 | 267 |
| Tsp009596_8_120-Tsp004851_2891_2779 | Trans-NAT | 289 | 4895 | 194 | 4104 | 95 | 791 |
| Tsp009540_1_330-Tsp007371_2144_1813 | Trans-NAT | 288 | 672 | 74 | 211 | 214 | 461 |
| Tsp009540_1_330-Tsp008238_2243_1912 | Trans-NAT | 275 | 662 | 74 | 211 | 201 | 451 |
| Tsp009122_385_561-Tsp007410_5937_5761 | Trans-NAT | 264 | 1287 | 101 | 816 | 163 | 471 |
| Tsp002535_587_815-Tsp008589_369_141 | Trans-NAT | 251 | 658 | 93 | 219 | 158 | 439 |
| Tsp009122_385_561-Tsp000235_6168_5992 | Trans-NAT | 251 | 1198 | 101 | 816 | 150 | 382 |
| Tsp008905_425_819-Tsp009235_453_59 | Trans-NAT | 247 | 814 | 2 | 2 | 245 | 812 |
| Tsp006225_203_282-Tsp009549_126_47 | Trans-NAT | 239 | 1512 | 49 | 176 | 190 | 1336 |
| Tsp009926_9_162-Tsp005171_1420_1267 | Trans-NAT | 238 | 1683 | 151 | 1274 | 87 | 409 |
| Tsp009235_1_256-Tsp007445_1324_1069 | Trans-NAT | 233 | 641 | 134 | 498 | 99 | 143 |
| Tsp009235_1_256-Tsp008216_1267_1012 | Trans-NAT | 232 | 640 | 134 | 498 | 98 | 142 |
| Tsp009072_1_248-Tsp007445_1324_1077 | Trans-NAT | 231 | 639 | 133 | 497 | 98 | 142 |
| Tsp009072_1_248-Tsp008216_1267_1020 | Trans-NAT | 231 | 639 | 133 | 497 | 98 | 142 |
| Tsp009342_29_185-Tsp007998_2255_2098 | Trans-NAT | 229 | 815 | 136 | 597 | 93 | 218 |
| Tsp004978_1_286-Tsp008966_418_133 | Trans-NAT | 226 | 2214 | 39 | 62 | 187 | 2152 |
| Tsp009540_73_330-Tsp008237_1160_903 | Trans-NAT | 221 | 552 | 65 | 175 | 156 | 377 |
| Tsp007201_623_966-Tsp006566_995_652 | Trans-NAT | 218 | 616 | 22 | 31 | 196 | 585 |
| Tsp004920_159_315-Tsp007478_216_56 | Trans-NAT | 217 | 711 | 163 | 624 | 54 | 87 |
| Tsp006762_308_579-Tsp007202_5042_4761 | Trans-NAT | 216 | 458 | 63 | 148 | 153 | 310 |
| Tsp006762_316_579-Tsp006565_5013_4740 | Trans-NAT | 213 | 455 | 61 | 146 | 152 | 309 |
| Tsp006762_316_579-Tsp008993_403_130 | Trans-NAT | 213 | 455 | 61 | 146 | 152 | 309 |
| Tsp009715_34_121-Tsp007677_180_92 | Trans-NAT | 213 | 1692 | 36 | 97 | 177 | 1595 |
| Tsp006762_308_579-Tsp009408_390_109 | Trans-NAT | 209 | 445 | 63 | 148 | 146 | 297 |
| Tsp009926_2_166-Tsp007257_1265_1101 | Trans-NAT | 209 | 1437 | 156 | 1280 | 53 | 157 |
| Tsp009926_2_166-Tsp007410_1493_1329 | Trans-NAT | 209 | 1437 | 156 | 1280 | 53 | 157 |
| Tsp009103_4_87-Tsp009867_84_1 | Trans-NAT | 208 | 1507 | 156 | 1382 | 52 | 125 |
| Tsp000009_1_170-Tsp007910_168_1 | Trans-NAT | 206 | 1361 | 190 | 1343 | 16 | 18 |
| Tsp000009_1_170-Tsp003140_165_1 | Trans-NAT | 205 | 1370 | 190 | 1343 | 15 | 27 |
| Tsp009620_1_381-Tsp008290_831_451 | Trans-NAT | 203 | 486 | 171 | 444 | 32 | 42 |
| Tsp009620_33_381-Tsp009773_900_553 | Trans-NAT | 199 | 477 | 166 | 437 | 33 | 40 |
| Tsp009342_1_185-Tsp008035_1168_985 | Trans-NAT | 198 | 749 | 154 | 682 | 44 | 67 |
| Tsp007885_2040_2128-Tsp008937_93_5 | Trans-NAT | 191 | 1363 | 18 | 50 | 173 | 1313 |
| Tsp008905_394_819-Tsp009072_483_59 | Trans-NAT | 188 | 610 | 2 | 2 | 186 | 608 |
| Tsp004911_1_108-Tsp000585_572_465 | Trans-NAT | 187 | 3260 | 187 | 3260 | 0 | 0 |
| Tsp004920_165_311-Tsp005430_210_60 | Trans-NAT | 186 | 660 | 160 | 617 | 26 | 43 |
| Tsp007201_623_966-Tsp006565_1121_778 | Trans-NAT | 184 | 471 | 22 | 31 | 162 | 440 |
| Tsp007201_623_966-Tsp010142_575_232 | Trans-NAT | 184 | 471 | 22 | 31 | 162 | 440 |
| Tsp006811_121_326-Tsp008100_202_1 | Trans-NAT | 183 | 2427 | 150 | 2371 | 33 | 56 |
| Tsp001358_41_159-Tsp007376_395_277 | Trans-NAT | 177 | 1086 | 74 | 828 | 103 | 258 |
| Tsp006811_121_319-Tsp007910_205_8 | Trans-NAT | 176 | 2404 | 148 | 2366 | 28 | 38 |
| Tsp009715_1_101-Tsp007251_1254_1154 | Trans-NAT | 176 | 494 | 75 | 257 | 101 | 237 |
| Tsp005010_1424_1522-Tsp009334_109_11 | Trans-NAT | 175 | 1382 | 126 | 1229 | 49 | 153 |
| Tsp005010_1424_1522-Tsp010050_109_11 | Trans-NAT | 175 | 1382 | 126 | 1229 | 49 | 153 |
| Tsp005147_1280_1550-Tsp005183_2756_2486 | Trans-NAT | 174 | 675 | 6 | 8 | 168 | 667 |
| Tsp009620_5_381-Tsp007722_2180_1804 | Trans-NAT | 174 | 448 | 171 | 444 | 3 | 4 |
| Tsp009620_5_381-Tsp008323_1149_773 | Trans-NAT | 174 | 448 | 171 | 444 | 3 | 4 |
| Tsp009511_83_190-Tsp007898_108_1 | Trans-NAT | 170 | 357 | 75 | 141 | 95 | 216 |
| Tsp009511_83_190-Tsp009262_108_1 | Trans-NAT | 170 | 357 | 75 | 141 | 95 | 216 |
| Tsp009715_34_128-Tsp008399_441_346 | Trans-NAT | 167 | 1023 | 68 | 766 | 99 | 257 |
| Tsp007224_67_267-Tsp010092_247_44 | Trans-NAT | 166 | 1581 | 121 | 1461 | 45 | 120 |
| Tsp007224_77_276-Tsp006565_5209_5007 | Trans-NAT | 166 | 1581 | 121 | 1461 | 45 | 120 |
| Tsp007224_77_276-Tsp008993_599_397 | Trans-NAT | 166 | 1581 | 121 | 1461 | 45 | 120 |
| Tsp007224_77_276-Tsp010076_361_159 | Trans-NAT | 166 | 1581 | 121 | 1461 | 45 | 120 |
| Tsp001219_2_288-Tsp005354_559_273 | Trans-NAT | 165 | 393 | 129 | 348 | 36 | 45 |
| Tsp009736_1_152-Tsp000949_1042_891 | Trans-NAT | 165 | 484 | 36 | 54 | 129 | 430 |
| Tsp007477_73_219-Tsp009882_409_261 | Trans-NAT | 164 | 497 | 92 | 322 | 72 | 175 |
| Tsp009736_194_323-Tsp000468_428_298 | Trans-NAT | 160 | 953 | 36 | 259 | 124 | 694 |
| Tsp007200_13_303-Tsp006566_942_652 | Trans-NAT | 159 | 495 | 21 | 30 | 138 | 465 |
| Tsp001219_7_288-Tsp005205_554_273 | Trans-NAT | 154 | 404 | 126 | 344 | 28 | 60 |
| Tsp002772_2223_2448-Tsp005481_223_3 | Trans-NAT | 152 | 462 | 9 | 12 | 143 | 450 |
| Tsp008496_114_201-Tsp004415_426_338 | Trans-NAT | 151 | 9574 | 30 | 43 | 121 | 9531 |
| Tsp009783_27_225-Tsp007647_2000_1802 | Trans-NAT | 149 | 533 | 14 | 21 | 135 | 512 |
| Tsp008153_1_145-Tsp009094_180_36 | Trans-NAT | 147 | 308 | 71 | 181 | 76 | 127 |
| Tsp009736_194_323-Tsp000456_653_523 | Trans-NAT | 146 | 922 | 36 | 259 | 110 | 663 |
| Tsp005198_290_456-Tsp000949_2219_2054 | Trans-NAT | 141 | 338 | 0 | 0 | 141 | 338 |
| Tsp009729_1_86-Tsp008557_339_255 | Trans-NAT | 139 | 439 | 97 | 313 | 42 | 126 |
| Tsp006813_85_283-Tsp007910_205_8 | Trans-NAT | 135 | 1586 | 107 | 1548 | 28 | 38 |
| Tsp006813_85_283-Tsp003140_202_8 | Trans-NAT | 134 | 1595 | 107 | 1548 | 27 | 47 |
| Tsp008965_1_88-Tsp007715_1381_1294 | Trans-NAT | 134 | 1033 | 28 | 57 | 106 | 976 |
| Tsp005433_25_107-Tsp006225_324_243 | Trans-NAT | 133 | 518 | 87 | 411 | 46 | 107 |
| Tsp001358_41_161-Tsp005800_1946_1826 | Trans-NAT | 131 | 982 | 74 | 828 | 57 | 154 |
| Tsp010160_152_402-Tsp008905_677_425 | Trans-NAT | 127 | 329 | 125 | 327 | 2 | 2 |
| Tsp007200_13_303-Tsp006565_1068_778 | Trans-NAT | 125 | 350 | 21 | 30 | 104 | 320 |
| Tsp007200_13_303-Tsp010142_522_232 | Trans-NAT | 125 | 350 | 21 | 30 | 104 | 320 |
| Tsp010162_298_560-Tsp009940_386_120 | Trans-NAT | 125 | 1265 | 117 | 1254 | 8 | 11 |
| Tsp010160_1_146-Tsp008216_1267_1122 | Trans-NAT | 121 | 221 | 74 | 147 | 47 | 74 |
| Tsp008153_4_145-Tsp005015_1449_1308 | Trans-NAT | 119 | 267 | 71 | 181 | 48 | 86 |
| Tsp008153_146_280-Tsp005118_685_551 | Trans-NAT | 116 | 435 | 81 | 368 | 35 | 67 |
| Tsp009511_83_190-Tsp001668_108_1 | Trans-NAT | 109 | 204 | 75 | 141 | 34 | 63 |
| Tsp007484_268_429-Tsp005147_3388_3227 | Trans-NAT | 107 | 999 | 99 | 991 | 8 | 8 |
| Tsp009820_4_113-Tsp001636_2750_2641 | Trans-NAT | 106 | 834 | 44 | 85 | 62 | 749 |
| Tsp008214_1_112-Tsp007310_378_267 | Trans-NAT | 105 | 314 | 103 | 312 | 2 | 2 |
| Tsp009601_211_322-Tsp005183_2756_2645 | Trans-NAT | 105 | 440 | 0 | 0 | 105 | 440 |
| Tsp008153_146_280-Tsp007958_571_437 | Trans-NAT | 103 | 395 | 81 | 368 | 22 | 27 |
| Tsp008214_1_110-Tsp008458_438_329 | Trans-NAT | 102 | 293 | 100 | 291 | 2 | 2 |
| Tsp009103_199_273-Tsp009495_258_184 | Trans-NAT | 101 | 693 | 72 | 640 | 29 | 53 |
| Tsp009511_539_698-Tsp004924_2640_2480 | Trans-NAT | 101 | 373 | 39 | 252 | 62 | 121 |
| Tsp005878_674_849-Tsp007382_1877_1702 | Trans-NAT | 99 | 3025 | 68 | 2978 | 31 | 47 |
| Tsp009736_1_151-Tsp000831_769_622 | Trans-NAT | 99 | 461 | 36 | 54 | 63 | 407 |
| Tsp008966_314_391-Tsp001522_666_589 | Trans-NAT | 98 | 428 | 52 | 268 | 46 | 160 |
| Tsp010038_2_287-Tsp007382_1928_1643 | Trans-NAT | 98 | 1998 | 54 | 1936 | 44 | 62 |
| Tsp001358_41_159-Tsp000847_395_277 | Trans-NAT | 97 | 868 | 74 | 828 | 23 | 40 |
| Tsp005409_25_107-Tsp006225_324_243 | Trans-NAT | 96 | 185 | 50 | 78 | 46 | 107 |
| Tsp010162_3_185-Tsp004924_4710_4528 | Trans-NAT | 90 | 214 | 67 | 176 | 23 | 38 |
| Tsp001358_41_159-Tsp008736_404_286 | Trans-NAT | 89 | 861 | 74 | 828 | 15 | 33 |
| Tsp004346_622_755-Tsp004365_222_89 | Trans-NAT | 89 | 171 | 24 | 45 | 65 | 126 |
| Tsp010038_2_287-Tsp010091_435_146 | Trans-NAT | 89 | 1982 | 54 | 1936 | 35 | 46 |
| Tsp005063_335_461-Tsp001020_656_530 | Trans-NAT | 87 | 921 | 4 | 5 | 83 | 916 |
| Tsp009783_30_225-Tsp008196_2345_2150 | Trans-NAT | 85 | 262 | 14 | 21 | 71 | 241 |
| Tsp008872_184_258-Tsp009103_273_199 | Trans-NAT | 84 | 654 | 12 | 14 | 72 | 640 |
| Tsp005082_60_140-Tsp000949_1174_1094 | Trans-NAT | 70 | 235 | 0 | 0 | 70 | 235 |
| Tsp010038_38_287-Tsp008140_1050_801 | Trans-NAT | 69 | 1956 | 54 | 1936 | 15 | 20 |
| Tsp010038_38_287-Tsp008483_1080_831 | Trans-NAT | 69 | 1956 | 54 | 1936 | 15 | 20 |
| Tsp007468_362_480-Tsp000847_167_49 | Trans-NAT | 68 | 241 | 66 | 239 | 2 | 2 |
| Tsp007940_4_143-Tsp008049_441_302 | Trans-NAT | 66 | 161 | 23 | 37 | 43 | 124 |
| Tsp009540_78_232-Tsp004921_427_273 | Trans-NAT | 65 | 155 | 45 | 131 | 20 | 24 |
| Tsp007468_362_465-Tsp007939_305_202 | Trans-NAT | 63 | 236 | 62 | 235 | 1 | 1 |
| Tsp007468_362_468-Tsp007310_356_250 | Trans-NAT | 63 | 236 | 62 | 235 | 1 | 1 |
| Tsp004346_622_755-Tsp008778_222_89 | Trans-NAT | 62 | 117 | 24 | 45 | 38 | 72 |
| Tsp004346_622_755-Tsp009010_222_89 | Trans-NAT | 62 | 117 | 24 | 45 | 38 | 72 |
| Tsp007468_362_468-Tsp008059_167_61 | Trans-NAT | 62 | 235 | 62 | 235 | 0 | 0 |
| Tsp007661_1130_1216-Tsp004346_896_810 | Trans-NAT | 61 | 92 | 59 | 90 | 2 | 2 |
| Tsp007916_80_169-Tsp008635_244_155 | Trans-NAT | 56 | 235 | 4 | 6 | 52 | 229 |
| Tsp002029_169_323-Tsp005354_3447_3292 | Trans-NAT | 51 | 87 | 0 | 0 | 51 | 87 |
| Tsp005198_192_456-Tsp000857_1610_1346 | Trans-NAT | 46 | 144 | 2 | 2 | 44 | 142 |
| Tsp003256_1_326-Tsp009417_326_1 | Trans-NAT | 42 | 84 | 11 | 20 | 31 | 64 |
| Tsp007223_146_240-Tsp008919_107_13 | Trans-NAT | 39 | 258 | 0 | 0 | 39 | 258 |
| Tsp009833_1_207-Tsp003256_326_120 | Trans-NAT | 38 | 62 | 35 | 59 | 3 | 3 |
| Tsp005578_1_304-Tsp009417_326_23 | Trans-NAT | 36 | 78 | 11 | 20 | 25 | 58 |
| Tsp009452_1_207-Tsp003256_326_120 | Trans-NAT | 36 | 60 | 33 | 57 | 3 | 3 |
| Tsp005063_335_463-Tsp008457_566_438 | Trans-NAT | 35 | 118 | 5 | 6 | 30 | 112 |
| Tsp007710_2_276-Tsp007959_2411_2129 | Trans-NAT | 35 | 70 | 0 | 0 | 35 | 70 |
| Tsp003256_1_326-Tsp009558_329_1 | Trans-NAT | 31 | 63 | 11 | 20 | 20 | 43 |
| Tsp005578_1_304-Tsp009558_329_23 | Trans-NAT | 30 | 62 | 11 | 20 | 19 | 42 |
| Tsp007477_146_219-Tsp009943_363_288 | Trans-NAT | 28 | 58 | 0 | 0 | 28 | 58 |
| Tsp008929_36_206-Tsp009452_377_207 | Trans-NAT | 28 | 30 | 21 | 22 | 7 | 8 |
| Tsp006163_201_375-Tsp006226_246_72 | Trans-NAT | 26 | 35 | 4 | 5 | 22 | 30 |
| Tsp006163_201_375-Tsp009075_246_72 | Trans-NAT | 26 | 35 | 4 | 5 | 22 | 30 |
| Tsp006163_201_375-Tsp010123_246_72 | Trans-NAT | 25 | 34 | 4 | 5 | 21 | 29 |
| Tsp010162_408_502-Tsp005160_97_3 | Trans-NAT | 24 | 38 | 22 | 35 | 2 | 3 |
| Tsp005198_169_272-Tsp000831_3005_2902 | Trans-NAT | 23 | 49 | 2 | 2 | 21 | 47 |
| Tsp003214_3070_3552-Tsp008466_483_1 | Trans-NAT | 20 | 26 | 12 | 16 | 8 | 10 |
| Tsp002479_46_271-Tsp009417_251_26 | Trans-NAT | 19 | 42 | 0 | 0 | 19 | 42 |
| Tsp002479_46_287-Tsp009558_251_10 | Trans-NAT | 19 | 42 | 0 | 0 | 19 | 42 |
| Tsp003160_75_201-Tsp009075_246_120 | Trans-NAT | 16 | 18 | 1 | 1 | 15 | 17 |
| Tsp009808_32_158-Tsp009075_246_120 | Trans-NAT | 16 | 18 | 1 | 1 | 15 | 17 |
| Tsp003160_75_201-Tsp010123_246_120 | Trans-NAT | 15 | 17 | 1 | 1 | 14 | 16 |
| Tsp005045_105_262-Tsp009671_541_384 | Trans-NAT | 15 | 15 | 3 | 3 | 12 | 12 |
| Tsp005045_80_262-Tsp001636_1817_1635 | Trans-NAT | 15 | 15 | 3 | 3 | 12 | 12 |
| Tsp009808_32_158-Tsp010123_246_120 | Trans-NAT | 15 | 17 | 1 | 1 | 14 | 16 |
| Tsp009055_329_501-Tsp010014_335_163 | Trans-NAT | 14 | 15 | 6 | 7 | 8 | 8 |
| Tsp009374_466_638-Tsp010014_335_163 | Trans-NAT | 14 | 15 | 6 | 7 | 8 | 8 |
| Tsp003237_5_165-Tsp006225_197_36 | Trans-NAT | 12 | 13 | 11 | 12 | 1 | 1 |
| Tsp007710_2_136-Tsp005110_281_146 | Trans-NAT | 12 | 15 | 0 | 0 | 12 | 15 |
| Tsp009256_60_249-Tsp010008_229_39 | Trans-NAT | 11 | 11 | 2 | 2 | 9 | 9 |
| Tsp009886_142_228-Tsp004831_652_565 | Trans-NAT | 11 | 20 | 0 | 0 | 11 | 20 |
| Tsp008845_41_449-Tsp008981_462_53 | Trans-NAT | 10 | 12 | 0 | 0 | 10 | 12 |
| Tsp009034_123_240-Tsp010008_261_144 | Trans-NAT | 10 | 10 | 1 | 1 | 9 | 9 |
| Tsp001256_2185_2479-Tsp008371_1014_720 | Trans-NAT | 8 | 8 | 1 | 1 | 7 | 7 |
| Tsp001204_1443_1978-Tsp001205_1498_963 | Trans-NAT | 7 | 9 | 3 | 4 | 4 | 5 |
| Tsp007710_2_174-Tsp005059_2768_2588 | Trans-NAT | 7 | 9 | 0 | 0 | 7 | 9 |
| Tsp008845_6_449-Tsp009380_453_9 | Trans-NAT | 7 | 8 | 0 | 0 | 7 | 8 |
| Tsp000355_137_215-Tsp000521_222_144 | Trans-NAT | 5 | 5 | 5 | 5 | 0 | 0 |
| Tsp007232_906_996-Tsp010064_91_1 | Trans-NAT | 5 | 5 | 4 | 4 | 1 | 1 |
| Tsp008845_6_354-Tsp007921_981_633 | Trans-NAT | 5 | 6 | 0 | 0 | 5 | 6 |
| Tsp001751_62_136-Tsp007614_550_476 | Trans-NAT | 4 | 5 | 0 | 0 | 4 | 5 |
| Tsp001751_62_136-Tsp009794_277_203 | Trans-NAT | 4 | 5 | 0 | 0 | 4 | 5 |
| Tsp004452_1_179-Tsp004416_611_436 | Trans-NAT | 4 | 4 | 1 | 1 | 3 | 3 |
| Tsp004452_1_179-Tsp007548_611_436 | Trans-NAT | 4 | 4 | 1 | 1 | 3 | 3 |
| Tsp004452_1_179-Tsp008094_596_421 | Trans-NAT | 4 | 4 | 1 | 1 | 3 | 3 |
| Tsp007887_104_181-Tsp004902_833_756 | Trans-NAT | 4 | 4 | 2 | 2 | 2 | 2 |
| Tsp007887_104_181-Tsp005865_1952_1875 | Trans-NAT | 4 | 4 | 2 | 2 | 2 | 2 |
| Tsp002268_2370_2592-Tsp008774_249_27 | Trans-NAT | 3 | 3 | 0 | 0 | 3 | 3 |
| Tsp002749_161_246-Tsp008194_447_362 | Trans-NAT | 3 | 5 | 3 | 5 | 0 | 0 |
| Tsp002749_161_246-Tsp008284_1076_991 | Trans-NAT | 3 | 5 | 3 | 5 | 0 | 0 |
| Tsp004452_1_179-Tsp009069_371_196 | Trans-NAT | 3 | 3 | 1 | 1 | 2 | 2 |
| Tsp005013_968_1113-Tsp004891_196_49 | Trans-NAT | 3 | 3 | 0 | 0 | 3 | 3 |
| Tsp005013_970_1113-Tsp002168_194_49 | Trans-NAT | 3 | 3 | 0 | 0 | 3 | 3 |
| Tsp006225_1_129-Tsp008677_132_4 | Trans-NAT | 3 | 4 | 1 | 1 | 2 | 3 |
| Tsp007710_2_137-Tsp008174_349_211 | Trans-NAT | 3 | 4 | 0 | 0 | 3 | 4 |
| Tsp008845_6_449-Tsp008930_797_353 | Trans-NAT | 3 | 3 | 0 | 0 | 3 | 3 |
| Tsp009034_115_221-Tsp009559_237_131 | Trans-NAT | 3 | 3 | 1 | 1 | 2 | 2 |
| Tsp009256_179_265-Tsp005291_4358_4272 | Trans-NAT | 3 | 3 | 2 | 2 | 1 | 1 |
| Tsp009256_180_265-Tsp008579_130_45 | Trans-NAT | 3 | 3 | 2 | 2 | 1 | 1 |
| Tsp004255_1501_1670-Tsp008894_279_110 | Trans-NAT | 2 | 3 | 2 | 3 | 0 | 0 |
| Tsp005198_169_250-Tsp005011_2743_2662 | Trans-NAT | 2 | 2 | 2 | 2 | 0 | 0 |
| Tsp006366_728_1109-Tsp006368_389_8 | Trans-NAT | 2 | 2 | 2 | 2 | 0 | 0 |
| Tsp007887_104_181-Tsp007725_626_549 | Trans-NAT | 2 | 2 | 2 | 2 | 0 | 0 |
| Tsp007887_1_90-Tsp005144_3013_2924 | Trans-NAT | 2 | 2 | 0 | 0 | 2 | 2 |
| Tsp008241_766_886-Tsp009942_121_1 | Trans-NAT | 2 | 4 | 0 | 0 | 2 | 4 |
| Tsp008995_1_81-Tsp007069_191_111 | Trans-NAT | 2 | 2 | 1 | 1 | 1 | 1 |
| Tsp008995_1_81-Tsp007548_611_531 | Trans-NAT | 2 | 2 | 1 | 1 | 1 | 1 |
| Tsp008995_1_81-Tsp007986_377_297 | Trans-NAT | 2 | 2 | 1 | 1 | 1 | 1 |
| Tsp008995_1_81-Tsp009069_371_291 | Trans-NAT | 2 | 2 | 1 | 1 | 1 | 1 |
| Tsp009034_114_212-Tsp008579_237_139 | Trans-NAT | 2 | 2 | 1 | 1 | 1 | 1 |
| Tsp000373_59_214-Tsp008188_166_11 | Trans-NAT | 1 | 1 | 0 | 0 | 1 | 1 |
| Tsp000397_74_246-Tsp008772_222_50 | Trans-NAT | 1 | 2 | 0 | 0 | 1 | 2 |
| Tsp001280_1_83-Tsp001288_83_1 | Trans-NAT | 1 | 1 | 0 | 0 | 1 | 1 |
| Tsp001764_1865_1981-Tsp009241_486_370 | Trans-NAT | 1 | 1 | 1 | 1 | 0 | 0 |
| Tsp002013_458_582-Tsp002017_610_486 | Trans-NAT | 1 | 1 | 0 | 0 | 1 | 1 |
| Tsp002774_90_222-Tsp007274_2130_1998 | Trans-NAT | 1 | 1 | 0 | 0 | 1 | 1 |
| Tsp003355_69_269-Tsp008680_201_1 | Trans-NAT | 1 | 1 | 1 | 1 | 0 | 0 |
| Tsp003671_1_263-Tsp008733_269_17 | Trans-NAT | 1 | 1 | 0 | 0 | 1 | 1 |
| Tsp004061_1184_1305-Tsp008806_122_1 | Trans-NAT | 1 | 1 | 0 | 0 | 1 | 1 |
| Tsp004161_144_228-Tsp008878_131_47 | Trans-NAT | 1 | 1 | 0 | 0 | 1 | 1 |
| Tsp005103_736_882-Tsp003114_356_210 | Trans-NAT | 1 | 1 | 0 | 0 | 1 | 1 |
| Tsp005708_25_102-Tsp009032_78_1 | Trans-NAT | 1 | 1 | 1 | 1 | 0 | 0 |
| Tsp006345_113_326-Tsp008249_214_1 | Trans-NAT | 1 | 1 | 1 | 1 | 0 | 0 |
| Tsp007843_2004_2091-Tsp004765_1196_1110 | Trans-NAT | 1 | 1 | 0 | 0 | 1 | 1 |
| Tsp007886_154_334-Tsp008266_278_97 | Trans-NAT | 1 | 1 | 1 | 1 | 0 | 0 |
| Tsp009857_49_151-Tsp008184_1082_981 | Trans-NAT | 1 | 1 | 1 | 1 | 0 | 0 |
| Tsp009999_121_240-Tsp002061_300_181 | Trans-NAT | 1 | 1 | 0 | 0 | 1 | 1 |
| Tsp009999_121_240-Tsp004891_306_187 | Trans-NAT | 1 | 1 | 0 | 0 | 1 | 1 |
| Tsp010030_238_316-Tsp007934_966_888 | Trans-NAT | 1 | 1 | 1 | 1 | 0 | 0 |
| Tsp010030_238_317-Tsp001742_2030_1951 | Trans-NAT | 1 | 1 | 1 | 1 | 0 | 0 |
| Tsp010030_238_317-Tsp001778_884_805 | Trans-NAT | 1 | 1 | 1 | 1 | 0 | 0 |
